# Exploring the Interactions Between Methyl Methacrylate Polymers and the Bacterial Outer Membrane via Coarse-Grained Molecular Dynamics Simulations

**DOI:** 10.1021/acs.jcim.6c00729

**Published:** 2026-04-07

**Authors:** Eduardo R. Almeida, Vinicius Firmino dos Santos, Madeleine Ramstedt, Thereza A. Soares

**Affiliations:** † Department of Chemistry, FFCLRP, 28133 University of São Paulo, Ribeirão Preto, SP 14040-901, Brazil; ‡ Department of Chemistry, 8075 Umeå University, Umeå 901 87, Sweden; § Hylleraas Centre for Quantum Molecular Sciences, University of Oslo, Oslo 0315, Norway

## Abstract

Polymer brush coatings offer a promising strategy for combating bacterial adhesion and biofilm formation in medical devices. However, a detailed molecular understanding of how different brush chemistries interact with bacterial membranes remains incomplete. In this study, we use coarse-grained steered molecular dynamics and umbrella sampling simulations to investigate the interaction and translocation process of four methyl methacrylate-derived polymers: pDMAEMA (weak cationic), pMETAC (strong cationic), pMEDSAH (zwitterionic), and pSPMA (anionic), through a bacterial outer membrane (OM) model of*Escherichia coli*. The simulations reveal a four-step translocation process: approach, adhesion, permeation, and internalization, characterized by distinct thermodynamic and kinetic signatures. Cationic polymers exhibit a pronounced favorable adhesion with the OM surface, especially with the saccharide inner core domain of the LPS molecules, which is mainly attributed to their favorable electrostatic interactions. The dragging of LPS units to the inner leaflet of the bacterial OM was also distinguished in the translocation of these positively charged polymers. In contrast, zwitterionic and anionic polymers show less favorable adhesion, consistent with their antifouling behavior. This approach provides a computational framework to resolve the free-energy landscapes and structural perturbations associated with polymer–membrane interactions at molecular detail, including the prediction of kinetically unfavorable processes, such as the permeation and internalization of the polymer in the intracellular region of the membrane. These results offer mechanistic insights into how hydration, charge, and polymer structure influence bacterial membrane interactions, advancing the molecular design of antifouling and antibacterial surface coatings.

## Graphical abstract

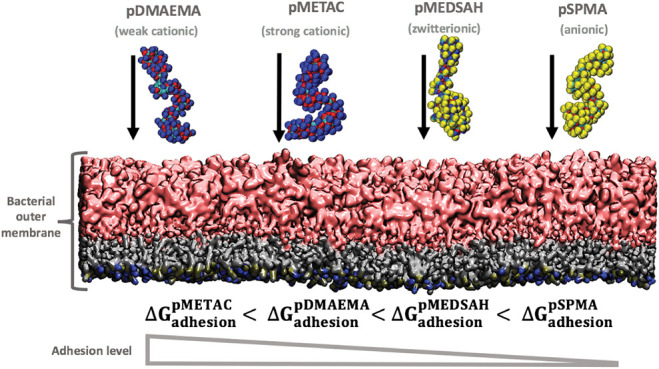

## 1 Introduction

Molecular brushes have been widely explored as antiadhesive and antibacterial coatings for the protection of surfaces of medical devices against bacterial infections. These brush systems can have a variety of chemical compositions, thus exhibiting a wide range of different chemical and physical properties that may be tailored for various purposes.^1–4^ Their biomedical relevance can therefore be adjusted to give systems not only with mechanical, thermal, and chemical stability but also with biocompatibility, antibacterial, and antiadhesive properties, even in complex biological media such as blood, serum, and saliva.^4–7^ In this work, we have focused on hydrophilic polyelectrolyte brushes that can be divided into strong polyelectrolytes, carrying charge at all pH ranges, and weak polyelectrolytes, whose charge depends on the pH of the surrounding solution, giving rise to switchable surfaces.^8^ Polyelectrolyte brushes of interest for applications within antiadhesive or antibacterial surfaces often carry zwitterionic, anionic, or cationic groups, where mainly the cationic ones include examples of both weak and strong polyelectrolytes.^4,9^ It has been established that polycationic brushes present a bactericidal mode of action due to their strong adhesion to the bacterial cell envelope that can destabilize the bacterial outer membrane (OM) inducing the leakage of ions and other species.^4,10,11^ However, the molecular mechanism for this is not well established, and several scenarios have been presented.^4,9^

Zwitterionic and anionic brushes generally exhibit antifouling activities since they repel bacterial adhesion and subsequent biofilm formation.^4,12^ Specifically, the repelling effect of zwitterionic polymers is attributed to their high hydrophilicity, which is expected to lead to the formation of tightly bounded solvation shells, and thus prevent their interactions with bacteria and other foulants.^13–15^ The repelling activity of anionic brushes has been attributed to a balance between the electrostatic repulsion upon interaction with the negatively charged bacterial surfaces and their hydrophilicity that provides a strong hydration layer.^4,16^ Thus, the antifouling property of anionic brushes is influenced by the presence of cations in the system since they may neutralize the anionic groups. These negatively charged brushes may take part in ion exchange reactions affecting their physicochemical properties, and it is not well known if such ion exchange may influence their interactions with the lipopolysaccharide (LPS) region of the OM in Gram-negative bacterial cells. In summary, the experimental results have demonstrated that the antifouling and antibacterial behavior of polymer brush-based coatings can be modulated by varying the inclusion of charged units in these systems.

Previously, computational simulations of molecular brushes were performed to investigate the interactions modulating the absorption of lysozyme^17,18^ and amino acid side-chain analogs.^19^ It was shown, through the use of molecular dynamics (MD) and Monte Carlo (MC) simulations, that zwitterionic polymer brushes exhibit stronger and more stable surface hydration than PEG, resulting in reduced lysozyme adsorption.^17,20^ This hydration pattern was assigned to the increased ordering of interfacial water structures and dipole networks with zwitterionic groups, whose orientation and interactions are regulated by the zwitterion type and the carbon spacer length within the brush architecture.^17,18^ MD simulations of three amino acid side-chain analogs onto the polymer brushes poly­(carboxybetaine methacrylate) (pCBMA) and poly­(2-hydroxyethyl methacrylate) (pHEMA) indicated that the nonpolar amino acid analog was attracted to pHEMA but repelled by pCBMA, whereas the charged amino acid analogs (propionate and butylammonium) were repelled by both brushes.^18^ The stronger interaction with water and steric repulsion of the solvation shell were the key factors responsible for the broader antifouling performance of the zwitterionic pCBMA in contrast to the more variable behavior of hydrophilic, nonzwitterionic pHEMA.^19^ A key conclusion from these studies is that antifouling performance correlates with the strength of interfacial hydration; polymer brushes exhibiting stronger surface hydration more effectively resist protein adsorption. This hydration layer serves as a physical and energetic barrier to protein adsorption, underscoring its critical role in antifouling mechanisms.

While significant insights into protein adsorption onto polymer brushes have been gained through simulations, the interactions between the OM of Gram-negative bacteria and polymer brushes remain underexplored. There are a multitude of reasons for this, but perhaps the most important is the greater complexity of the membrane that composes the bacterial cell wall structure.^21^ Unlike individual proteins, bacterial surfaces exhibit considerable heterogeneity and variations in composition, topology, charge distribution, and hydrophobicity, complicating accurate representation in simulations.^22–25^ Concerning the structure, the bacterial OM is an asymmetrical membrane composed of phospholipids in the inner leaflet and lipopolysaccharides (LPSs) in the outer leaflet.^26,27^ More specifically, the inner leaflet is composed of 75% of 1,2-dipalmitoyl-*sn*-glycero-3-phosphatidylethanolamine (DPPE), 25% of 1,2-dipalmitoyl-*sn*-glycero-3-phosphatidylglycerol (DPPG), and 1%–5% of 1′,3′-bis­(1,2-diacyl-*sn*-glycero-3-phospho)-*sn*-glycerol or cardiolipin, whereas the outer leaflet is mainly made of rough LPSs.^26^

Computational modeling complements experimental measurements by providing molecular-resolution access to the binding locus and the physical drivers of adhesion, such as electrostatic pairing, ion mediation, and local dehydration/rehydration. Moreover, free-energy calculations allow us to separate thermodynamic favorability (e.g., adhesion) from kinetically hindered processes (e.g., permeation), which are difficult to disentangle experimentally. This is particularly relevant for Gram-negative OM, where the heterogeneous LPS architecture can create strong, spatially specific interaction motifs. In this work, we employed steered molecular dynamics (SMD) and umbrella sampling (US) simulations to investigate the translocation of four classes of methyl methacrylate-derived polymers across the Gram-negative bacterial OM at the coarse-grained (CG) level. We focus on a family of methacrylate-based brushes that share a common backbone but differ in their ionic functionality, spanning weak and strong cationic (pDMAEMA, pMETAC), anionic (pSPMA), and zwitterionic (pMEDSAH) groups, so as to enable a chemistry-controlled comparison.^4^ These chemistries are widely used as antibacterial and antifouling coatings and provide a systematic test bed in which differences in membrane interactions can be attributed primarily to charge type and hydration. In addition, methacrylate brushes have an available revised MARTINI 3 coarse-grained parametrization,^27^ supporting consistent simulations across these polymer classes within a single validated force-field framework. The resulting free-energy profiles map out the thermodynamic landscape underlying the interactions between antifouling and antibacterial polymers and a bacterial OM model. The calculated free energy and force profiles can reveal the roles of electrostatics, hydration, and membrane remodeling in modulating antibacterial and antifouling properties.

## 2 Methods

In order to study the polymer brush–bacterial OM interactions, we employed a set of methyl-methacrylate-based polymer brushes: poly­(*N*,*N*-dimethyl laminoethyl methacrylate) (pDMAEMA), poly­(2-(methacryloyloxy)­ethyl)-trimethylammonium chloride (pMETAC), poly­(3-sulfopropyl methacrylate potassium) (pSPMA), and poly­([2-(methacryloyloxy)­ethyl]­dimethyl-(3-sulfopropyl)­ammonium) (pMEDSAH). The CG MD simulations of these polymers composed of 96 monomers per chain were performed using a revised parameter set of the MARTINI 3 force field (FF)^28^ for methyl-methacrylate-based polymer brushes.^27^ The choice of 96 monomers per chain follows our previous atomistic and CG simulations of methacrylate polyelectrolyte brushes, where this chain length yielded stable brush conformations and swelling behavior.^27,29^ This polymer chain length is suitable for probing the approach/adhesion to the bacterial OM while keeping the cost of steered MD and umbrella sampling tractable. The representation of the CG beads assigned to the chemical structures of the monomers that compose these four polymers is shown in Figure 1.

**Figure 1:**
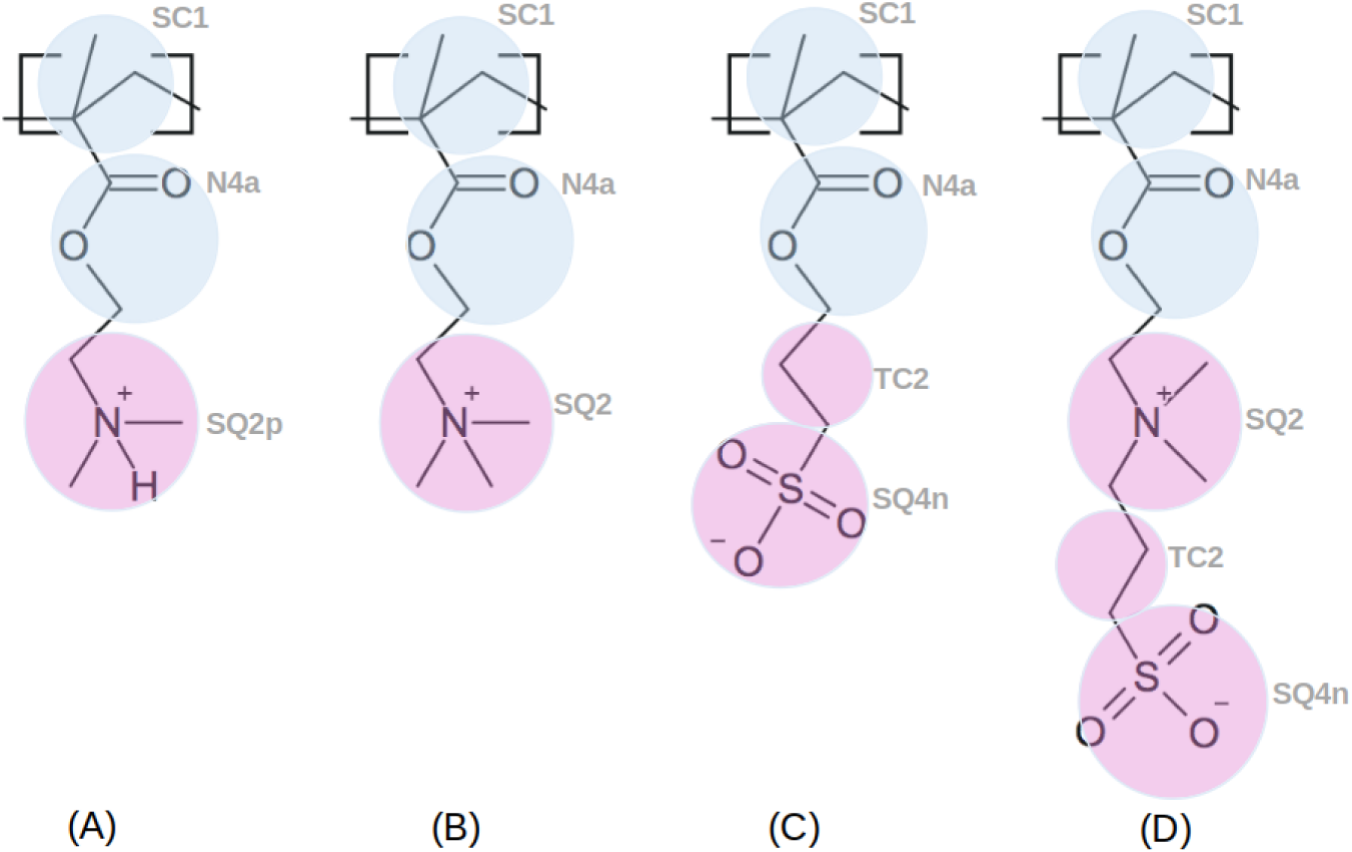
Structures of the monomers that compose the four polymers studied in this work: poly­(*N*,*N*-dimethyl laminoethyl methacrylate) (pDMAEMA) (A), poly­(2-(methacryloyloxy)­ethyl)-trimethylammonium chloride (pMETAC) (B), poly­(3-sulfopropyl methacrylate potassium) (pSPMA) (C), and poly­([2-(methacryloyloxy)­ethyl]­dimethyl-(3-sulfopropyl)­ammonium) (pMEDSAH) (D). The black lines represent the atomistic structures, and the blue and pink spheres refer to the coarse-grained structures. The symbols in gray represent the bead types as defined in the MARTINI 3 force field.^28^ The beads SC1, N4a, SQ2p, SQ2, TC2, and SQ4n indicate a small bead for linear alkanes, a regular bead for esters, a small bead for alkyl dimethylammonium (monovalent ion), a regular bead for tetramethylammonium (monovalent ion), a tiny bead for branched alkanes, and a small bead for sulfonates (monovalent ion), respectively. The beads SC1 and N4a have been modified for polymer brushes as described in ref 27.

The selected polymers were chosen for their key chemical properties relevant to antibacterial and antifouling applications, such as a weak cationic brush that shows pH responsiveness (pDMAEMA), a strong polycationic brush (pMETAC), a brush with zwitterionic nature (pMEDSAH), and a strong anionic polyelectrolyte brush (pSPMA). The main chemical and physical properties of these polymers are presented in Table S1. We simulated fully protonated pDMAEMA in order to compare its behavior with that of the strong cationic pMETAC. The CG bead selection, refinement, and validation against experimental data are discussed in more detail somewhere else.^27^

In a polymer brush, the free chain ends at the outermost region of the brush mediate the first interactions with external biological interfaces, such as bacterial membranes. These distal segments experience reduced packing constraints and exhibit dynamics more akin to free polymer chains than to those embedded deeply within the brush.^30^ At maximum length, the simulated polymers could reach 24 nm (totally extended), though chain flexibility, thermal motion, and solvation make the actual end-to-end distance in solution significantly shorter, depending on conditions like solvent, temperature, and ionic strength. Instead, the worm-like chain model predicts average lengths of 8–10 nm, which are also consistent with the values previously calculated from MD simulations of polymer brushes.^27,29^ As many studies have utilized brush coatings within the 13–50 nm range,^31^ the simulated polymer chains are expected to exhibit conformational dynamics closer to a free polymer chain rather than a strand in the brush regime.^32^ Moreover, the approach with a free chain allows for a semiquantitative exploration of the energetics and structural pathways governing polymer–membrane interactions, which would be technically exceedingly challenging for a full brush model, especially for systems as heterogeneous as the bacterial OM. Because full polymer translocation through the Gram-negative OM is not expected to occur spontaneously under physiological conditions, we do not interpret our simulations as predicting spontaneous permeation. Instead, we use this translocation pathway as a conceptual and computational framework, combining SMD with US to obtain a controlled free-energy landscape along a defined reaction coordinate. The resulting wells and barriers enable a systematic thermodynamic analysis of polymer interactions with distinct membrane regions and have been widely used in computational studies of membrane biophysics and drug delivery.^33–35^ These free-energy features are directly relevant to antibacterial and antiadhesive behavior: weakly bound polymers facing large insertion barriers are expected to form reversible, short-lived contacts, whereas deeper minima and smaller barriers promote stronger, more persistent association, while also providing quantitative insight into insertion propensity and local membrane perturbation.

For the bacterial OM model, we used the MARTINI 3 FF^28^ for the treatment of phospholipids (Figure 2B–C) and an extension of this CG FF to represent the *Escherichia coli* LPS structure with the R1-type core (Figure 2A).^36^ The rough LPS was selected for our membrane model since it is the most common form of this endotoxin found in notable concentration in Gram-negative bacteria.^37^ We modeled the inner leaflet of the OM as made of 75% DPPE and 25% DPPG. While cardiolipin has been reported at low abundance in the inner leaflet of Gram-negative envelopes (∼1–5% of total lipids), its fraction and leaflet distribution are strongly species- and condition-dependent, and it is typically associated with the inner membrane rather than the LPS-exposed outer leaflet that governs the surface properties studied here.^38,39^ Because cardiolipin can also form lateral microdomains, explicitly including it would introduce additional assumptions about localization and clustering and substantially increase sampling requirements. We therefore used a minimal DPPE/DPPG mixture to capture the dominant zwitterionic/anionic balance of the phospholipid leaflet while focusing on the polymer approach and adhesion at the OM surface. Water molecules were represented using the MARTINI 3 FF Tiny Bead (TW) model to improve the coarse-grain description of the hydration of the bacterial membrane, whereas the Ca^2+^ and Cl^–^ ions were modeled as beads for divalent (D) and monovalent (Q) ions, respectively. No positional restraints were applied to ions at any stage; instead, convergence and stability were assessed via long OM equilibration (6 μs), replica-averaged ion density and contact analyses, and standard umbrella sampling convergence diagnostics (window overlap histograms and block analysis). The composition of the bacterial OM model (Figure 2D) is shown in Table S2. The polymers and bacterial OM structures (Figure 2D) were equilibrated separately before the construction of the polymer–OM systems. Using these pre-equilibrated structures, we built four main systems by positioning the polymers in the bulk solvent at an average distance of 9.0 nm from the membrane center (Figure 2E). Henceforth, these four systems will be termed pDMAEMA–OM, pMETAC–OM, pMEDSAH–OM, and pSPMA–OM. A summary of all MD simulations conducted in this study is presented in Table 1.

**Figure 2:**
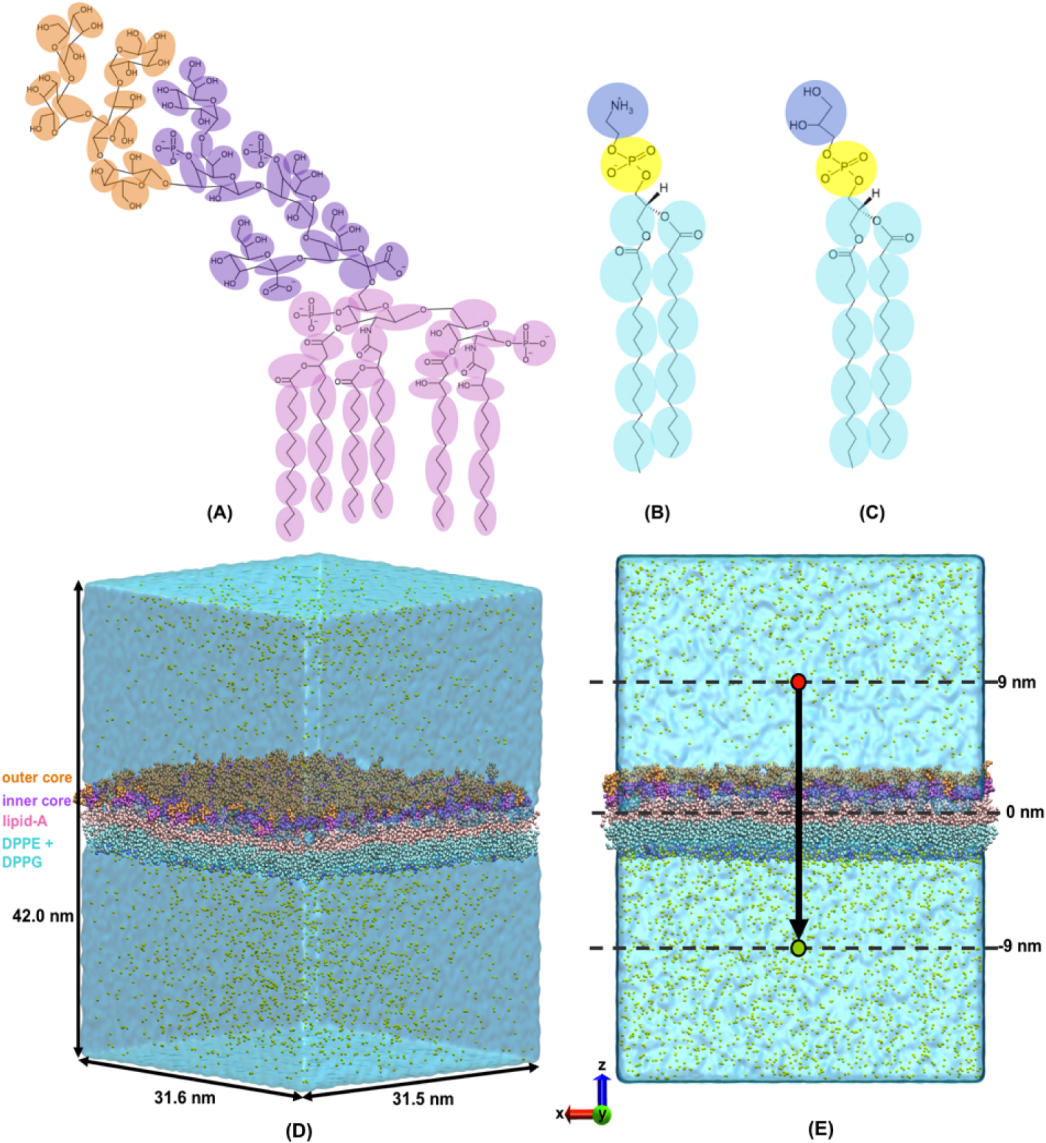
Chemical structures of the molecules (A, B, and C) that compose the bacterial outer membrane model for *Escherichia coli* (D and E) in both atomistic (structural formula in black lines) and coarse-grained resolutions (colored beads): lipopolysaccharide and its domains, namely lipid A (pink beads), inner core saccharides (purple beads), and outer core saccharides (orange beads) (A), 1,2-dipalmitoyl-*sn*-glycero-3-phosphoethanolamine (DPPE) (B), and 1,2-dipalmitoyl-*sn*-glycero-3-phospho-*rac*-glycerol (DPPG) (C) molecules. While the lipopolysaccharide molecules are located in the outer leaflet of the bacterial outer membrane, both DPPE and DPPG are found in the inner leaflet of this membrane. The solvent molecules are represented by tiny water (TW) beads (blue color) as defined in the MARTINI 3 force field.^28^ Definition of the reaction coordinate (black arrow) along the membrane normal (*z*-axis) for the steered molecular dynamics simulations of polymer translocation through the membrane (E).

**Table 1:** Simulated Systems and Simulation Times for Steered Molecular Dynamics and Umbrella Sampling

System	*N*_b_ ^a^	$N_{b}^{\mathrm{TW}}$ ^b^	*t*_eq_ /ns^c^	*t*_SMD_/ns^d^	t_US‑eq_/ns^e^	*t*_US‑pr_/ns^e^
pDMAEMA	76,764	76,380	1000	-	-	-
pMETAC	242,367	241,983	1000	-	-	-
pMEDSAH	205,020	204,540	1000	-	-	-
pSPMA	132,361	131,881	1000	-	-	-
OM	687,648	616,312	6000	-	-	-
pDMAEMA–OM	687,448	615,728	10	3 × 320	35 × 125	35 × 175
pMETAC–OM	687,490	615,770	10	3 × 320	36 × 125	36 × 175
pMEDSAH–OM	687,450	615,634	10	3 × 320	37 × 325	37 × 175
pSPMA–OM	687,470	615,654	10	3 × 320	36 × 300	36 × 200

a Total number of beads.

b Number of water beads.

c Equilibration time of the unbiased simulations.

d Simulation time for the steered molecular dynamics (SMD) of the translocation of the polymers (pDMAEMA, pMETAC, pMEDSAH, and pSPMA) through the bacterial outer membrane (OM). The term 3× indicates that these simulations were carried out in triplicate.

e Simulation time of the equilibration stage of the set of windows simulated with the umbrella sampling (US) method. For this column, the number before × refers to the total number of windows, while the number after the × indicates the simulation time per window. The first five systems refer to unbiased runs of the polymers and the bacterial OM. These systems were then combined into the last four systems corresponding to the biased MD simulations of the translocation processes of the polymers through the bacterial OM.

The first step of the unbiased CG MD simulations of the systems was energy minimization using the steepest descent method^40^ until the maximum force value was smaller than 10 kJ mol^–1^ nm^–1^. Regarding the polymers, the next step was the equilibration run under constant temperature and pressure (310 K and 1 bar) using the leapfrog algorithm^40^ with a time step of 10 fs and periodic boundary conditions in all directions. The initial velocities were generated according to a Maxwell distribution at 310 K. The temperature was kept constant with the v-rescale thermostat and coupling constants of 1 ps.^41^ The Berendsen barostat^42^ (coupling constant of 5 ps) was employed to maintain a constant pressure at 1 bar during the equilibration stages, whereas the Parrinello-Rahman barostat^43^ (coupling constants of 12 ps) was employed to maintain the same constant pressure (1 bar) during the production runs.

Concerning the bacterial OM model, the equilibration protocol consisted of four 5 ns stages in which the time step was progressively increased from 2 to 20 fs. This was followed by a 5 μs simulation using a 20 fs time step. The equilibrated membrane was then replicated four times to obtain the final OM model dimensions (31.6 nm × 31.5 nm × 42.0 nm; Table 1). Finally, the replicated system was simulated for an additional 1 μs in the NPT ensemble at 310 K and 1 bar using the v-rescale thermostat (coupling constant of 0.4 ps) and the Berendsen barostat (coupling constant of 5 ps). The membrane simulations were conducted under semi-isotropic pressure coupling (*x*/*y* plane and *z*-axis were allowed to vary independently) and an isothermal compressibility value of 3.0 × 10^–4^ bar^–1^. A cutoff distance of 1.2 nm was used to truncate the van der Waals (modeled using Lennard-Jones potential) and electrostatic interactions, and the reaction field approach^44^ was applied to treat the long-range electrostatic interactions with relative dielectric permittivity ε_r_ = 15 and ε_rf_ = ∞. Lennard-Jones potentials were shifted to zero at the cutoff distance. The neighbor lists were updated every 20 steps using the Verlet algorithm with a buffer tolerance of 0.005 kJ mol^–1^ ps^–1^.^45^

SMD simulations were employed to drive the complete translocation of the four polymers along the axis normal to the bacterial OM, using the center of mass (COM) pulling algorithm as implemented in GROMACS version 2019.4.^46^ The normal axis to the membrane was used as the reaction coordinate (Figure 2E). These simulations were conducted along an average distance of 18 nm with a pull rate of 0.0001 nm/ps and a force constant of 1000 kJ mol^–1^ nm^–2^.^47–49^ From these trajectories, we selected, on average, 36 frames with distance intervals of 0.3 nm along the reaction coordinate, which represent the position of the free polymer from the bulk solution toward the center of the bacterial OM (*z* = 0 nm; see the exact number of frames for each system in Table 1). These frames, also referred to as windows, were used as the starting configurations for the US simulations^50^ for each system, which were also performed using GROMACS version 2019.4.^46^ The simulation times for both the equilibration and production stages of the US windows are specified in Table 1. The potentials of mean forces (PMFs) for each system were computed by means of the weighted histogram analysis method (WHAM).^51^ The statistical errors of the PMFs were estimated using the bootstrap analysis implemented in the g_wham tool in the GROMACS suite.^52^ We note that a standard hysteresis analysis could not be performed because the present translocation process occurs across a compositionally asymmetric outer membrane for which the forward and reverse directions are not equivalent microscopic pathways. The asymmetric leaflets differ substantially in composition, electrostatics, hydration, ion binding, and the local mechanical response. Therefore, any discrepancy between forward and reverse profiles would conflate genuine membrane asymmetry with incomplete convergence and therefore would not provide an unambiguous hysteresis metric. We sought to assess convergence using direction-independent criteria, including the consistency among independent steered MD replicas used to generate the initial pathway, overlap of adjacent umbrella histograms, and the stability of the reconstructed PMFs under extended sampling and block-analysis-based error estimation. Furthermore, at the suggestion of one reviewer, additional short unbiased MD simulations were performed for the four polymers, each initialized at a distance of 1 nm from the outer membrane surface. In all cases, spontaneous adsorption to the membrane occurred within 100 ns or less, consistent with the favorable interfacial association identified from the SMD and US results. A flowchart explaining the computational workflow conducted herein was included in the Supporting Information (Figure S1). The structural analyses of the equilibration stages of the free polymers and the bacterial OM are presented in the Supporting Information (Figures S2 and S3, Tables S3 and S4).

## 3 Results and Discussion

In order to understand the interactions between different components of the bacterial OM and polymer chains at the molecular level, we employed SMD simulations to translocate the polymers through the equilibrated bacterial OM (Figure 2E). This translocation process can be depicted as a multistep process with four stages: approach, adhesion, permeation, and internalization. The approach stage occurs within the first 18 ns of simulation when the polymers diffuse from the bulk solvent to the bacterial OM surface. It is followed by the adhesion stage (18–34 ns), which corresponds to the polymer insertion into the polysaccharide core of the outer leaflet of the bacterial OM. The next stage is the permeation phase (34–112 ns) when the polymers reach the hydrophobic center of the membrane. The last stage of the mechanism is the internalization step when the polymer exits the bacterial OM into the solvent (112–320 ns). An in-depth molecular-level description of each step is presented in the next sections.

### 3.1 Effects of the Polymer Translocation on the Structure of the Bacterial OM

The mass density profiles of the bacterial OM for the polymer translocation process were computed to characterize its structural effect on the bacterial OM (Figure 3). It can be seen that despite localized and transient membrane defects (including local thinning and water penetration) during the permeation and internalization steps, the lamellar arrangement of the bacterial OM was not substantially affected by polymer translocation. The density profiles show that the approach (Figure 3A) and adhesion (Figure 3B) steps do not significantly alter the OM structure. Indeed, the most evident structural changes in the OM take place during the internalization (Figure 3D) step. In particular, the average mass density of 100 kg m^–3^ for the water at the center of the membrane located at 0 nm on the *z*-axis normal to the membrane (Figure 3D) shows the presence of solvent in this region after the permeation of the four polymers through the bacterial OM. These molecules have crossed the membrane as part of the polymer solvation shell or due to polymer-induced localized defects (local thinning and transient water penetration) in the OM. We will discuss the structural dynamics of polymer solvation in the next section.

**Figure 3:**
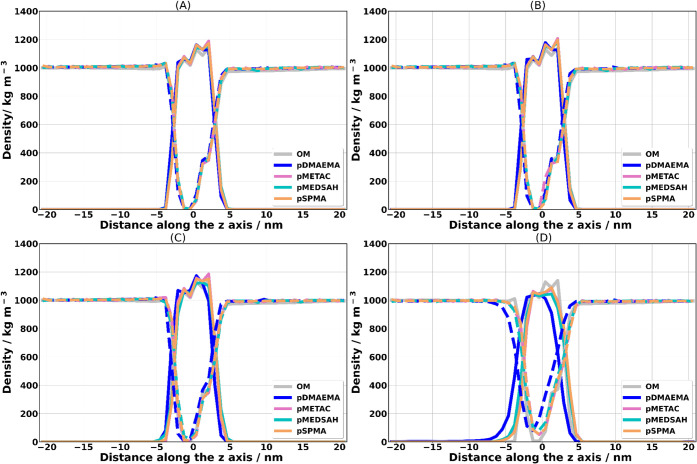
Mass density profiles of the bacterial outer membrane (OM) across the *z*-axis of the simulation box were calculated from the translocation of the polymers (pDMAEMA, pMETAC, pMEDSAH, and pSPMA) through the membrane. The profiles were calculated for each step of the translocation mechanisms: approach step (A), adhesion step (B), permeation step (C), and internalization step (D). The solid lines represent the profiles of the membrane, while the dashed lines refer to the solvent (water). The silver lines indicate the profile for the polymer-free bacterial OM. The polymers approach the membrane from the right to the left side of these profiles. Each profile was calculated as an average from three independent runs.

The carbohydrate moiety of the LPS membranes has previously been shown to be highly hydrated and charged due to the presence of the phosphate and carboxyl groups.^23,24,53–58^ For this reason, the structural stability of these systems is closely connected to the hydration pattern, the ionic valence, and the cross-linking interactions.^23,24,53–58^ It was previously shown that only divalent cations are able to stabilize the LPS-based aggregates in lamellar phases through cross-linking of phosphate groups from adjacent LPS molecules in the bacterial OM.^23,24,53–58^ Hence, we also computed the mass density profiles of these ions for each step of the translocation mechanism in order to probe the average position of the Ca^2+^, Na^+^, and Cl^–^ ions on the bacterial OM (Figure 4 and Figure S4).

**Figure 4:**
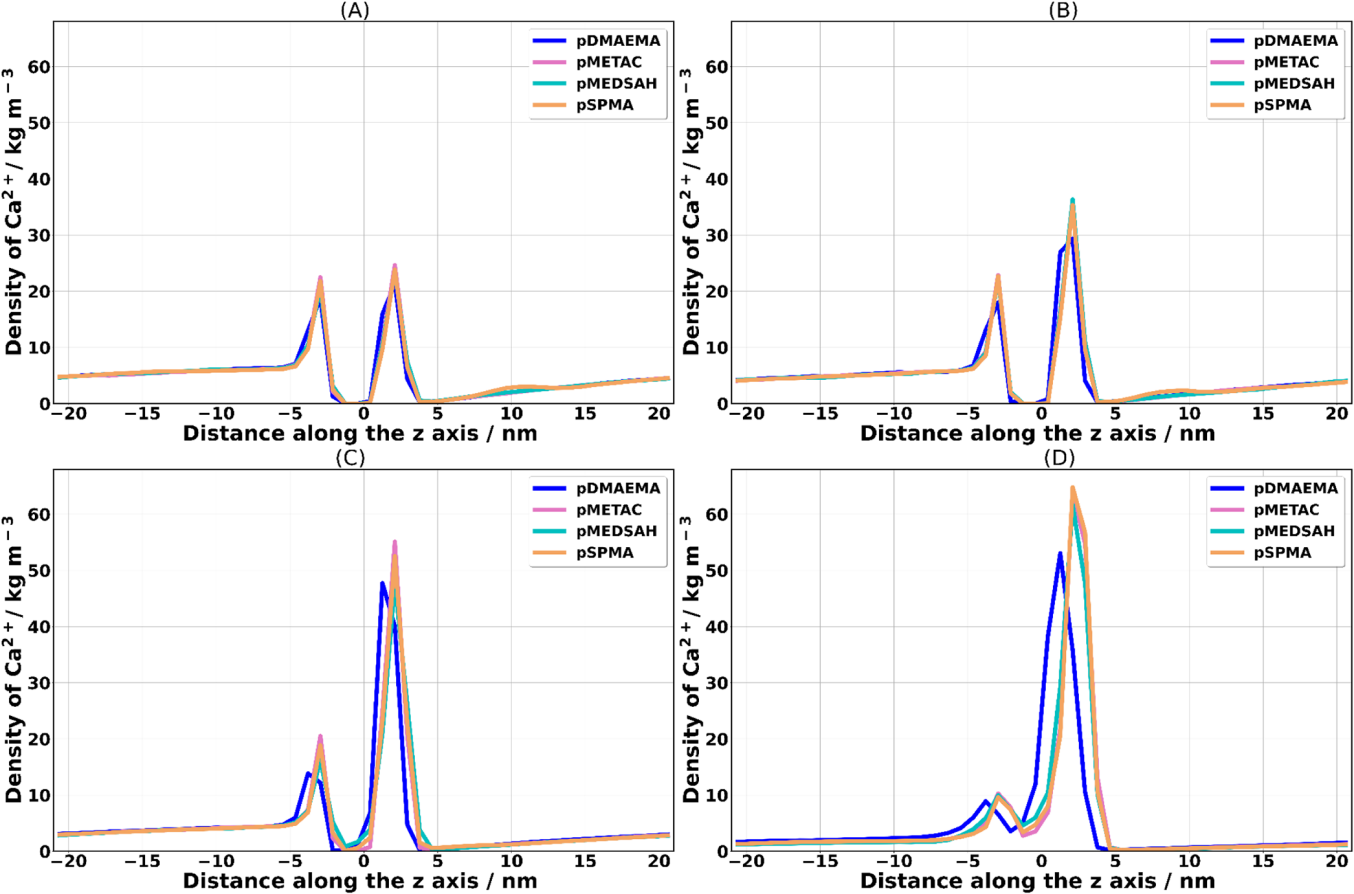
Mass density profiles of Ca^2+^ ions during the translocation process of the polymers (pDMAEMA, pMETAC, pMEDSAH, and pSPMA) through the normal axis (*z*-axis) to the bacterial outer membrane (OM): approach step (A), adhesion step (B), permeation step (C), and internalization step (D). Each profile was calculated as an average from three independent runs. The polymers approach the membrane from the right to the left side of these density profiles.

On average, the Ca^2+^ ions were found to be coordinated in the vicinity of the LPS molecules as well as in the region of the polar headgroups of the phospholipids (Figure 4). These ionic concentrations, expressed by the two main peaks, result from the favorable interactions between these divalent ions and the anionic species (LPS and the DPPG molecules), thereby confirming that the charged beads are able to capture the electrostatics of these systems in line with data from experiments and atomistic simulations.^23,24,53–58^ The most noticeable modification of these density profiles takes place in the permeation and internalization steps, when there is an increase in the intensity and a widening of the two main peaks, with a clear asymmetry of the Ca^2+^ density between the two regions in which there is a concentration of this divalent ion: the LPS region at about 2.5 nm from the center of the OM and the region of the polar headgroups of the phospholipids at about −2.5 nm from the center of the OM. Moreover, the internalization step of the polymers also evidenced an average density of about 4.0 kg m^–3^ for Ca^2+^ ions at the center of the membrane (Figure 4D). As we previously observed in the case of the solvent (Figure 3D), this behavior indicates that the polymer internalization also leads to a greater redistribution of the Ca^2+^ ions along the membrane and the penetration of these species into the membrane.

Regarding the Cl^–^ and Na^+^ ions, the mass density profiles reveal a preferential accumulation of Na^+^ ions within the LPS leaflet throughout all translocation stages of the pSPMA (Figure S4), though to a lesser extent than that observed for the Ca^2+^ ions (Figure 4). From the adhesion to the internalization step, the progressive increase in the intensity of the Na^+^ density peaks indicates a concentration of this species in the LPS leaflet of the bacterial OM, likely driven by the translocation of the anionic polymer (pSPMA), which carries coordinated Na^+^ ions along its backbone. However, the profiles indicate the absence of Na^+^ and Cl^–^ diffusion, on average, across the membrane from the extracellular to the intracellular region (Figure S4C–S4D). The increased Na^+^ accumulation in the LPS leaflet (Figure S4), together with the presence of Ca^2+^ ions (Figure 4), also promotes the local enrichment of Cl^–^ ions in this region, as evidenced by the rise in Cl^–^ density from the permeation to the internalization step (Figure S4C–S4D).

In order to specify the interactions of the polymers with the ions and lipids during the translocation process, we computed the average number of contacts between these species during the steps of the translocation mechanism (Table 2). The quantification of the contacts between polymers and ions shows that mainly the anionic polymer (pSPMA) dragged Ca^2+^ ions from the approach step until the internalization step, hence explaining the concentration of this divalent ion at the center of the OM (Figure 4D). The absence of contacts between Ca^2+^ and the cationic polymers (pDMAEMA and pMETAC) during the internalization step (Table 2), despite their nonzero density at the center of the membrane (Figure 4D), suggests that these ions are at least 0.6 nm away from the polymers. The decrease in the average number of pSPMA···Ca^2+^ contacts from 63.8 ± 1.7 during the approach and adhesion steps to 54.6 ± 2.1 during the permeation and internalization steps corresponds to an average loss of 9.2 contacts after crossing the center of the OM. A similar behavior was observed for the contacts between cationic polymers (pDMAEMA and pMETAC) and Cl^–^ ions. In particular, an average loss of 5.2 pMETAC···Cl^–^ contacts was observed from the approach to the internalization step of this polymer. The mass density profile broadening for the internalization step (Figure 3D) indicates the presence of lipids bound to the polymers, especially pDMAEMA, after exiting the membrane. The polymer-bound lipids are LPS molecules that make favorable electrostatic interactions with the positively charged polymers. The absence of contacts between polymers and phospholipids (DPPE and DPPG) points out that the LPS molecules remained interacting with the polymers during the translocation, thereby hindering interactions with other species. Furthermore, this absence of contacts between the polymers and the inner leaflet of the bacterial (OM) during the permeation and internalization steps suggests the opening of holes in the membrane during the arrival of the polymer in the intracellular medium.

**Table 2:** Average Number of Contacts between the Polymers (pDMAEMA, pMETAC, pMEDSAH, and pSPMA) and the Ions (Ca^2+^, Na^+^, and Cl^–^) and Lipids (LPS, DPPE, and DPPG) during the Translocation Mechanisms through the Bacterial Outer Membrane (OM)^a^

Systems	Polymer ···Ca^2+^	Polymer ···Na^+^	Polymer···Cl^–^	Polymer···LPS	Polymer ···DPPG	Polymer ···DPPE
	**Approach step**					
pDMAEMA–OM	0	0	2.4 ± 0.4	0	0	0
pMETAC–OM	0	0	22.9 ± 3.8	0	0	0
pMEDSAH–OM	4.7 ± 0.6	0	0	0	0	0
pSPMA–OM	62.4 ± 1.5	0	0	0	0	0
	**Adhesion step**					
pDMAEMA–OM	0	0	3.0 ± 0.8	0	0	0
pMETAC–OM	0	0	26.7 ± 8.9	0	0	0
pMEDSAH–OM	3.2 ± 0.4	0	0	59.2 ± 12.1	0	0
pSPMA–OM	65.2 ± 0.7	0	0	0	0	0
	**Permeation step**					
pDMAEMA–OM	0	0	2.7 ± 1.1	205.6 ± 15.5	0	0
pMETAC–OM	0	0	23.9 ± 6.4	137.5 ± 5.4	0	0
pMEDSAH–OM	3.2 ± 0.5	0	0	242.9 ± 17.7	0	0
pSPMA–OM	52.5 ± 1.6	0	0	113.9 ± 3.1	0	0
	**Internalization step**					
pDMAEMA–OM	0	0	3.2± 3.1	247.5 ± 45.4	0	0
pMETAC–OM	0	0	15.3 ± 2.5	132.9 ± 6.3	0	0
pMEDSAH–OM	4.4 ± 0.5	0	0	116.5 ± 11.0	0	0
pSPMA–OM	56.6 ± 1.4	0	0	96.8 ± 9.3	0	0

a All values were calculated as averages from three independent runs, considering a cutoff distance for contacts of 0.6 nm. The symbol ··· represents the interaction between two species.

### 3.2 Solvation of the Polymers during Their Translocation through the Bacterial OM

We have also investigated the solvent structure around the polymer chains during the translocation process through analyses of the radial distribution functions (*g*(*r*); Figure 5). The decrease in the intensity of the *g*(*r*) peaks between polymer and water molecules during the permeation (Figure 5C) and internalization (Figure 5D) steps results from the desolvation of the polymer chains, as expected upon polymer embedding into the membrane. The more intense *g*(*r*) peaks observed for pSPMA at the permeation (Figure 5C) and internalization (Figure 5D) steps indicate notable preservation of its solvation shell during these stages of the translocation. This strong hydration of pSPMA also suggests a lower interaction of this polymer with the OM, which is consistent with the expected electrostatic repulsion between these systems due to the anionic character of both pSPMA and LPS molecules. This observation is in close agreement with the established view of pSPMA as bacteria-repellent coatings.^4,59^ In contrast, the low intensities for pDMAEMA and pMETAC during the permeation (Figure 5C) and internalization (Figure 5D) steps indicate that the interactions between these polycationic structures and LPS molecules are dominant. The desolvation of both pDMAEMA and pMETAC at the internalization step is associated with the favorable adhesion of LPSs, which, in turn, shifted the solvation shells of these polymer–LPS systems, reducing the intensity of the TW–polymer interaction (Figure 5D).

**Figure 5:**
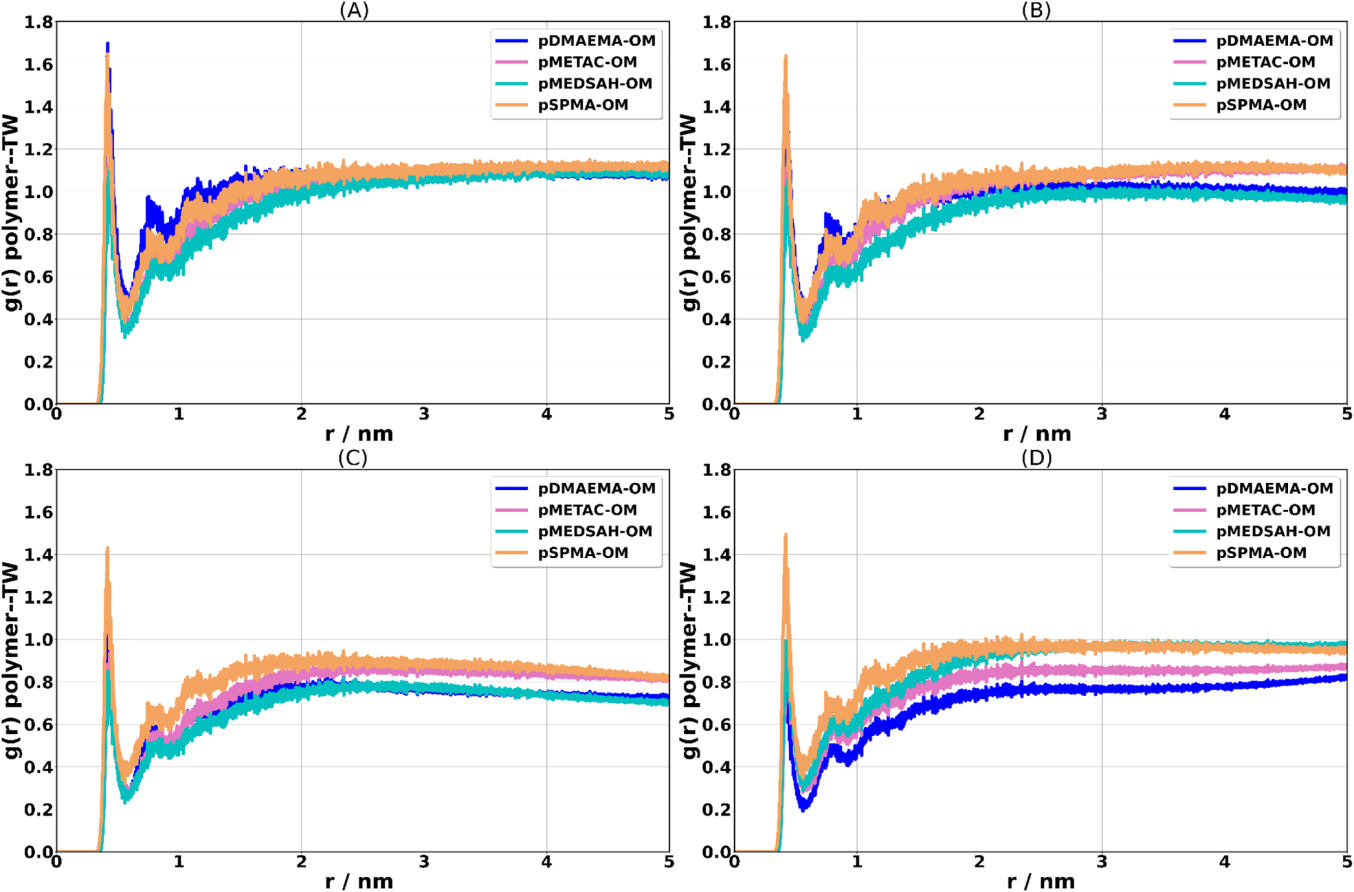
Radial distribution functions (*g*(*r*)) defined between the center of mass of the polymers and the water beads (TW) during the steps of the interaction mechanism with the bacterial outer membrane (OM): approach (A), adhesion (B), permeation (C), and internalization (D). Each *g*(*r*) was calculated as an average from three independent runs.

We have integrated *g*(*r*) curves (Figure 5) to determine the coordination numbers (CNs) of water beads (TW bead in the framework of the MARTINI 3 FF^28^) and provide a more quantitative analysis of the solvation structure surrounding the polymers throughout the translocation stages (Figure 6 and Table S5). The solvation shells of all four polymers decrease during the first three stages of the translocation process, especially during the permeation and internalization steps (Figure 6). During the adhesion step, there was a reduction of only 0.4% and 0.9% of the CN for the cationic polymer pMETAC and the anionic polymer pSPMA, respectively, while the zwitterionic pMEDSAH and the cationic pDMAEMA presented a CN reduction of 7.1% and 6.5%, respectively. In the permeation step, the CN decreases even more (25.5% for pDMAEMA, 21.8% for pMETAC, 22.7% for pMEDSAH, and 19.1% for pSPMA), confirming a greater desolvation of the zwitterionic polymer pMEDSAH and the cationic polymer pDMAEMA compared to one of the cationic pMETAC and anionic pSPMA species (Figure 6 and Table S5). These results, again, reveal a comparatively greater binding affinity of cationic polymers to the negatively charged bacterial OM during the last three stages of the translocation mechanism, especially at the internalization step, in which the CNs are similar to those in the permeation step. This result reinforces the strong interaction between these positively charged polymers and the LPS molecules, as already evidenced in Table 2.

**Figure 6:**
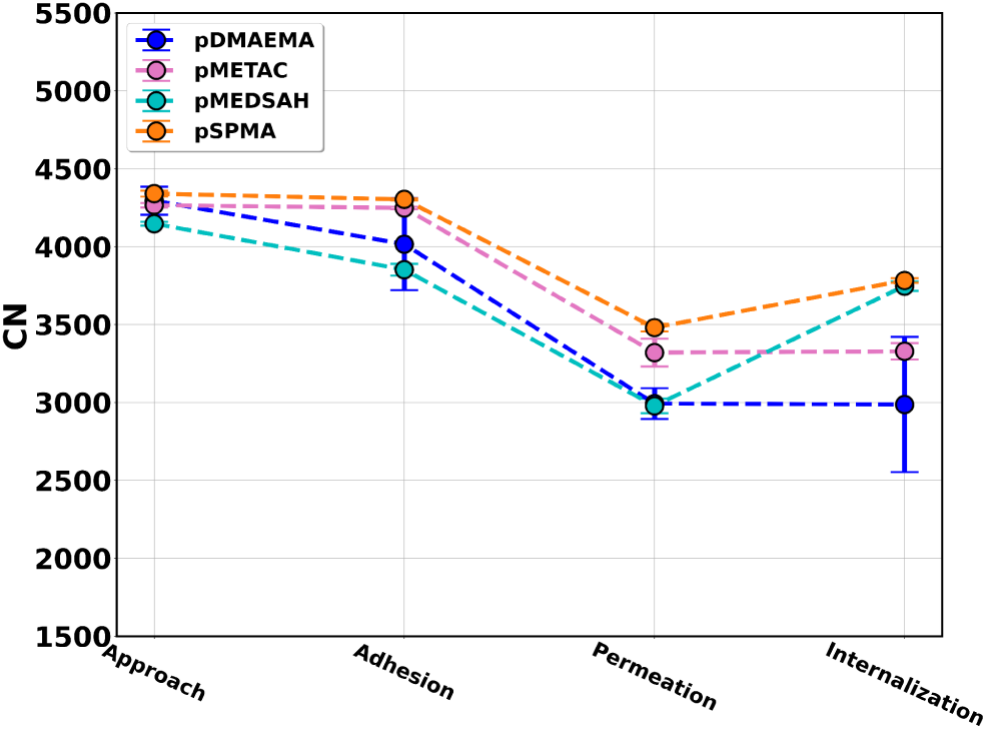
Coordination number (CN) of water beads (TW bead of the MARTINI 3 force field) at a 4.0 nm radial distance from the studied polymers (pDMAEMA, pMETAC, pMEDSAH, and pSPMA) during the steps of their translocation through the bacterial outer membrane (OM). Each value represents the average of three independent replicates.

At the internalization step, a higher hydration of zwitterionic (pMEDSAH) and anionic (pSPMA) polymers was observed (Figure 6, Table S5). Zwitterionic polymers have been previously shown to exhibit strong hydration through different techniques, e.g., contact angle measurements, quartz crystal microbalance (QCM), atomic force microscopy (AFM), X-ray photoelectron spectroscopy (XPS), and sum frequency generation (SFG) vibrational spectroscopy.^14,60–63^ In particular, SFG spectroscopy revealed the presence of a hydration layer at the polymer/water interface, which has been associated with decreased protein adsorption.^62,63^ Thus, the behavior of the CN for the pMEDSAH and especially pSPMA during the permeation and internalization steps is in line with the antifouling activity of zwitterionic and anionic polymers with respect to protein adhesion, and resulting from the strong surface hydration of these systems.^15,60–63^ It has been shown that proteins (e.g., BSA, fibrinogen, lysozyme) do not disrupt this hydration and that marine animals, such as mussels, are repelled from these surfaces even when mechanical forces are applied to promote attachment.^63^ When it comes to the pSPMA, the high CN during the adhesion, permeation, and internalization steps (Figure 6) also aligns with the preserved hydration of anionic brushes in contact with bacteria due to the electrostatic repulsion between these polymers and the surface of bacterial OMs.^4,64,65^

### 3.3 Force Profiles for the Polymer Translocation through the Bacterial OM

By using SMD simulations, we obtained the force profiles for the translocation of the four polymers across the bacterial OM, with the membrane normal serving as the reaction coordinate (Figure 7). The adhesion forces (*F*_adh_) and maximum forces (*F*_max_) were also computed from these simulations (Table 3). The *F*_adh_, defined as the force exerted on the polymer to induce its adhesion to the membrane surface, increases progressively as the four polymers interact with the bacterial OM (Figure 7 and Table 3). The cationic polymers (pDMAEMA and pMETAC) exhibit the highest *F*_adh_ values, exceeding those of pMEDSAH and pSPMA by an average of 110.45 kJ mol^–1^ nm^–1^ and 179.25 kJ mol^–1^ nm^–1^, respectively. This pattern may be attributed to the strong and favorable interactions between the positively charged polymers and the negatively charged LPS that compose the outer leaflet of the bacterial OM. Thus, greater forces were required for pulling pDMAEMA and pMETAC through the membrane. These findings are consistent with atomic force microscopy-based single-cell force spectroscopy measurements comparing the antimicrobial activity of three brushes (pMETAC, pSPMA, and neutral poly­(2-hydroxyethyl methacrylate) (PHEMA)).^31^ It has been reported that there is a strong interaction between the cationic pMETAC and the bacterial membrane.^12,31^

**Figure 7:**
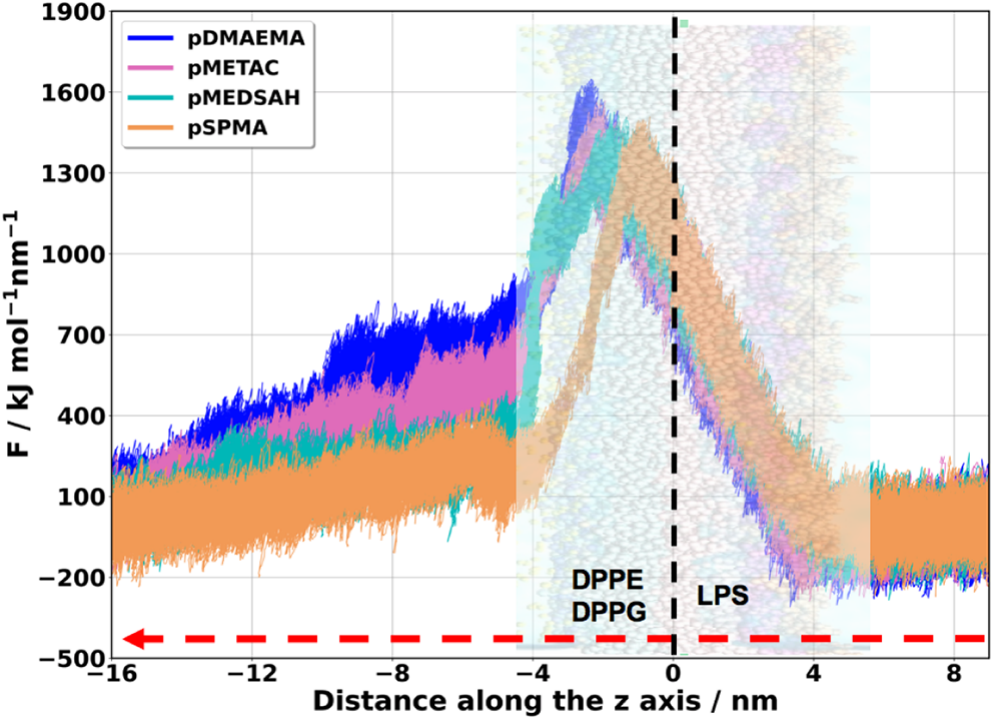
Temporal variation of the force applied (*F*) to the polymers (pDMAEMA, pMETAC, pMEDSAH, and pSPMA) during the steered molecular dynamics simulations (320 ns per simulation) of their translocation through the bacterial outer membrane (OM). Each curve represents the average of three independent replicates. The polymers approach the membrane from the left to the right side of these profiles. The dotted black line indicates the center of the bacterial OM, whereas the dotted red arrow indicates the direction of polymer translocation through the membrane.

**Table 3:** Adhesion Force (*F*_adh_) and Maximum Force (*F*_max_) Applied to the Free Polymers during Their Translocation Processes through the Bacterial Outer Membrane (OM) Model

System	*F*_adh_/kJ mol–^1^ nm^–1^ ^a^	*F*_max_/kJ mol–^1^ nm^–1^ ^b^
pDMAEMA–OM	236.6 ± 19.1	1940.0 ± 102.2
pMETAC–OM	292.9 ± 11.7	1855.7 ± 228.4
pMEDSAH–OM	154.3 ± 31.7	1762.9 ± 215.2
pSPMA–OM	85.5 ± 4.1	1704.3 ± 97.2

a Adhesion force collected from the steered molecular dynamics (SMD) simulations when the polymer reaches the membrane surface (at ∼4.4 nm in the reaction coordinate, see Figure 7).

b Maximum force collected from the SMD simulations. Each value represents the average of three independent replicates.

In contrast, the low *F*_adh_ values for pMEDSAH and pSPMA evidence weak interactions between these polymers and the surface of the bacterial OM. This behavior was expected due to the zwitterionic (pMEDSAH) and anionic (pSPMA) characteristics of these species that reduce the interactions with the OM surface. In spite of the large volume of these polymers compared to the cationic ones (Figure 1), these results demonstrate that the steric effect of the polymeric chains is less important than the intensity of the interactions during their translocation through the membrane. Furthermore, pSPMA exhibits a strong interaction with the solvent (Figure 5) and a large solvation shell, as evidenced by the CNs (Figure 6). This hydration shell effectively screened the polymer–membrane interactions, leading to a reduction in the force required to translocate the polymer along the membrane.

The highest force peaks in the force profiles are located at the center of the membrane, which demonstrates that polymer permeation was the most resistive step of the translocation mechanism through the OM (Figure 7). The resistance of this stage is mainly related to the steric effects imposed by the presence of the whole polymer structure, including its solvation shell (Figure 6), inside the membrane, which induces localized, transient membrane defects (local thinning and water penetration) during the permeation step. The maximum force (*F*_max_), which also reflects the binding affinity of a pulled molecule for its host,^66^ applied in the translocation of the four polymers through the bacterial OM has the largest values for pDMAEMA and pMETAC, indicating that these cationic systems have the highest binding affinity for the OM (Table 3). These favorable interactions between the cationic polymers and the OM, especially those involving the LPS, were also noticed by the number of contacts between these polymers and the LPS molecules during the permeation step (Table 2). The presence of these cationic polymers with adsorbed LPSs and the distinguished contact number with Cl^–^ ions at the center of the OM may have contributed to these force peaks during the permeation step, in addition to undoing noncovalent interactions during the translocation (Table 2, Table 3, and Figure 7). Conversely, the lowest *F*_max_ values were observed for the zwitterionic (pMEDSAH) and the anionic (pSPMA) polymers, which in turn indicates that these systems have the lowest binding affinity for the membrane (Table 3). The narrower force profiles, especially for the pSPMA, and the low values of forces for pulling pMEDSAH and pSPMA compared to the applied forces for the cationic polymers after crossing the center of the membrane (*z* = 0 in Figure 7) reflect the low affinity of these polymers for the bacterial OM. At last, despite the differences in the average *F*_max_ values, the high standard deviation values (Table 3) indicate that these differences are not significant.

### 3.4 Thermodynamics and Kinetics of the Translocation Processes

To describe the thermodynamics and kinetics of the translocation processes of the polymers through the bacterial OM, we have computed the potentials of mean force (PMFs) from the US simulations using the WHAM.^50,51^ Unlike the SMD simulations that considered the full reaction coordinate (Figure 2E and Figure 7), the calculation of the PMFs took into account only the translocation of polymers until the center of the bacterial OM, since this route is the most relevant for describing the surface effects related to the antibacterial and antifouling activities of the polymers. Thus, only the approach, adhesion, and the beginning of the permeation steps are thermodynamically represented in the PMFs. The sampling of the reaction coordinate along the *z*-axis of the membrane (Figure 2E) was confirmed by the overlap of the configuration histograms corresponding to the windows for the US method (Figure S5). Regarding convergence, the block analysis of the PMFs in Figure S6 supports the use of the simulation times cited in Table 1 for computing these free-energy profiles. The PMF curves superimposed on the membrane density profiles are presented in Figure 8, whereas the values of free energy of adhesion (Δ*G*_adh_) and the free-energy barrier $\left(\Delta G_{\mathrm{per}}^{‡}\right)$ with respect to the permeation of the polymers through the bacterial OM are shown in Table 4.

**Figure 8:**
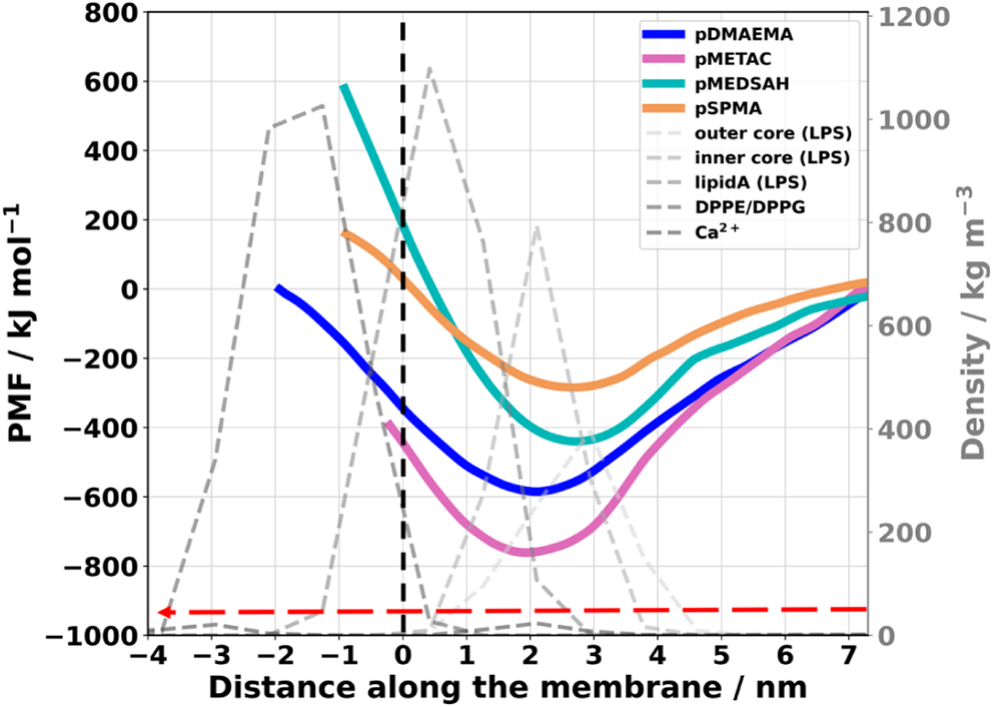
Potentials of mean force (PMFs) for the translocation processes of the polymers (pDMAEMA, pMETAC, pMEDSAH, and pSPMA) toward the center of the bacterial outer membrane (OM). The polymers approach the membrane from the left to the right side of these profiles, as indicated by the dotted red arrow. The dotted black line indicates the center of the bacterial OM. The grayscale curves depict the mass density profiles of the bacterial OM components (see Figure 2).

**Table 4:** Adhesion Free Energy (Δ*G*_adh_) for the Adsorption of the Polymers (pDMAEMA, pMETAC, pMEDSAH, and pSPMA) on the Surface of the Bacterial Outer Membrane (OM), and Free-Energy Barrier $\left(\Delta G_{\mathrm{per}}^{‡}\right)$ with Respect to the Partial Permeation of These Polymers Through the Same Membrane

System	Δ*G*_adh_/kJ mol^‑1^ ^a^	$\Delta G_{\mathrm{per}}^{‡}$ /kJ mol^‑1^ ^b^
pDMAEMA–OM	–585.6 ± 2.1	75.9 ± 5.4
pMETAC–OM	–761.3 ± 12.1	82.8 ± 12.1
pMEDSAH–OM	–440.4 ± 2.1	266.4 ± 4.4
pSPMA–OM	–284.4 ± 5.2	134.6 ± 7.2

a Free-energy minima obtained from the potentials of mean force (PMFs) calculated with the Umbrella Sampling (US) and Weighted Histogram Analysis Method (WHAM).

b The free-energy barriers were also collected from the PMFs as the energy difference between the free-energy minima and the free energy at the center of the bacterial OM (at *z* = 0 nm in the reaction coordinate).

The free-energy profiles show that the approach and adhesion steps were thermodynamically favorable for all polymers, especially for the cationic polymers (pDMAEMA and pMETAC), as also quantified by the associated negative values of Δ*G*_adh_ (Table 4). In particular, the cationic pMETAC exhibits an Δ*G*_adh_ that is 320.9 kJ mol^–1^ more negative than pMEDSAH and 476.9 kJ mol^–1^ than pSPMA, indicating substantially more favorable adhesion. The difference of 175.7 kJ mol^–1^ in the Δ*G*_adh_ between pMETAC and pDMAEMA reinforces the strong cationic character of pMETAC in contrast to the weak cationic behavior of pDMAEMA.^4,29^ By examining the superposition of the PMF energy minima of the cationic polymers (pDMAEMA and pMETAC) with the bacterial OM density profiles, it is possible to verify that the most favorable adhesion of these polymers takes place at the inner core domain (Figure 2) of the LPS molecules of the OM (at *z* = 2.0 nm in Figure 8). Specifically, these free-energy minima arise from favorable electrostatic interactions between the polycationic polymers and the saccharide moieties of the bacterial OM, particularly the anionic sugar residues 3-deoxy-d-manno-oct-2-ulopyranosonic acid (KDO) and l-glycero-d-manno-heptose (HEP), and the polycationic polymers. These results illustrate the pronounced electrostatic interaction between the positively charged polymers and the negatively charged surface of the bacterial OM, which plays an important role in the bacterial adhesion process to polycationic polymers. This is consistent with previous single-cell force spectroscopy measurements for the detachment of *Escherichia coli* from the pMETAC brush, which exhibited an average work for deadhesion (28 ± 9 nN nm) higher than the ones for the anionic (pSPMA) and neutral (pHEMA) brushes.^31^

Conversely, the less negative values of the PMFs and Δ*G*_adh_ for both pMEDSAH and pSPMA point out their more unfavorable adhesion to the bacterial OM compared to the cationic polymers (Figure 8, Table 4). Moreover, the adhesion process of pSPMA is thermodynamically less favorable than that of pMEDSAH by 156.0 kcal mol^–1^. These results are in agreement with the expected electrostatic repulsion between the anionic (pSPMA) and zwitterionic (pMEDSAH) polymers and the negatively charged region of the bacterial OM.^4,14^ The energy minima associated with the adhesion of both pSPMA and pMEDSAH are shifted toward the outer region of the bacterial membrane (at approximately *z* = 2.7 nm; Figure 8) compared to the minima observed for the adhesion of the cationic polymers. This shift also reflects a more repulsive adhesion of the zwitterionic (pMEDSAH) and anionic (pSPMA) polymers in the outer core domain (Figure 2) of the LPS molecules of the OM, which is the periphery of this membrane composed of glucose and galactose sugars.^36^ Single-cell force spectroscopy measurements have also characterized the interactions between the anionic pSPMA and *Escherichia coli* as significantly repulsive, with low detachment forces (0–100 pN).^31^ Additionally, the adhesion of the pMEDSAH to the bacterial OM is also consistent with single-cell force spectroscopy data showing the weak interaction between polyzwitterionic brushes and *Yersinia pseudotuberculosis*.^61^

When it comes to polymer permeation through the bacterial OM (at *z* = 0 nm in Figure 8), the high positive $\Delta G_{\mathrm{per}}^{‡}$ values indicate that this step is kinetically unfavorable for all systems, especially for pMEDSAH (Table 4). Such energy barriers indicate that the complete polymer translocation, including the internalization step, is not expected to occur spontaneously. This behavior also aligns with the high *F*_max_ peaks indicated in the SMD simulations (Table 3 and Figure 7). The average $\Delta G_{\mathrm{per}}^{‡}$ for the cationic polymers (79.4 ± 13.2 kcal mol^–1^) is about 187.0 kcal mol^–1^ and 55.2 kcal mol^–1^ lower than those of pMEDSAH and pSPMA, respectively. This result may also be related to the smaller molecular volume of the cationic polymers in comparison to those of the zwitterionic and anionic polymers (Figure 1), thereby inducing a smaller steric repulsion of the former during their permeation step through the membrane. In spite of these differences, the high energy barriers (Table 4) suggest that the polymer permeation and internalization are prohibited steps in the translocation mechanism of these systems.

### 3.5 Proposed Mechanism for Polymer Translocation across the Bacterial OM

Based on the analyses conducted so far, we provide a molecular-level description of the translocation mechanism of the four methyl methacrylate-derived polymers through the bacterial OM and relate these findings to their antifouling and antibacterial applications (Figure 9). The first step of the translocation mechanism was the approach step, in which the polymers were located up to about 6.0 nm from the membrane surface (Figure 9A). The free-energy profiles demonstrated that this step was thermodynamically favorable for all polymers, particularly for the cationic polymers (pDMAEMA and pMETAC; Figure 8). In addition to not affecting the global structure of the bacterial OM (Figure 3A), the approach stage was characterized, in general, by a progressive desolvation of the polymers at different levels according to their hydrophilicity and the interaction strength with the membrane, until they reached the bacterial OM surface during the adhesion step (Figure 6).

**Figure 9:**
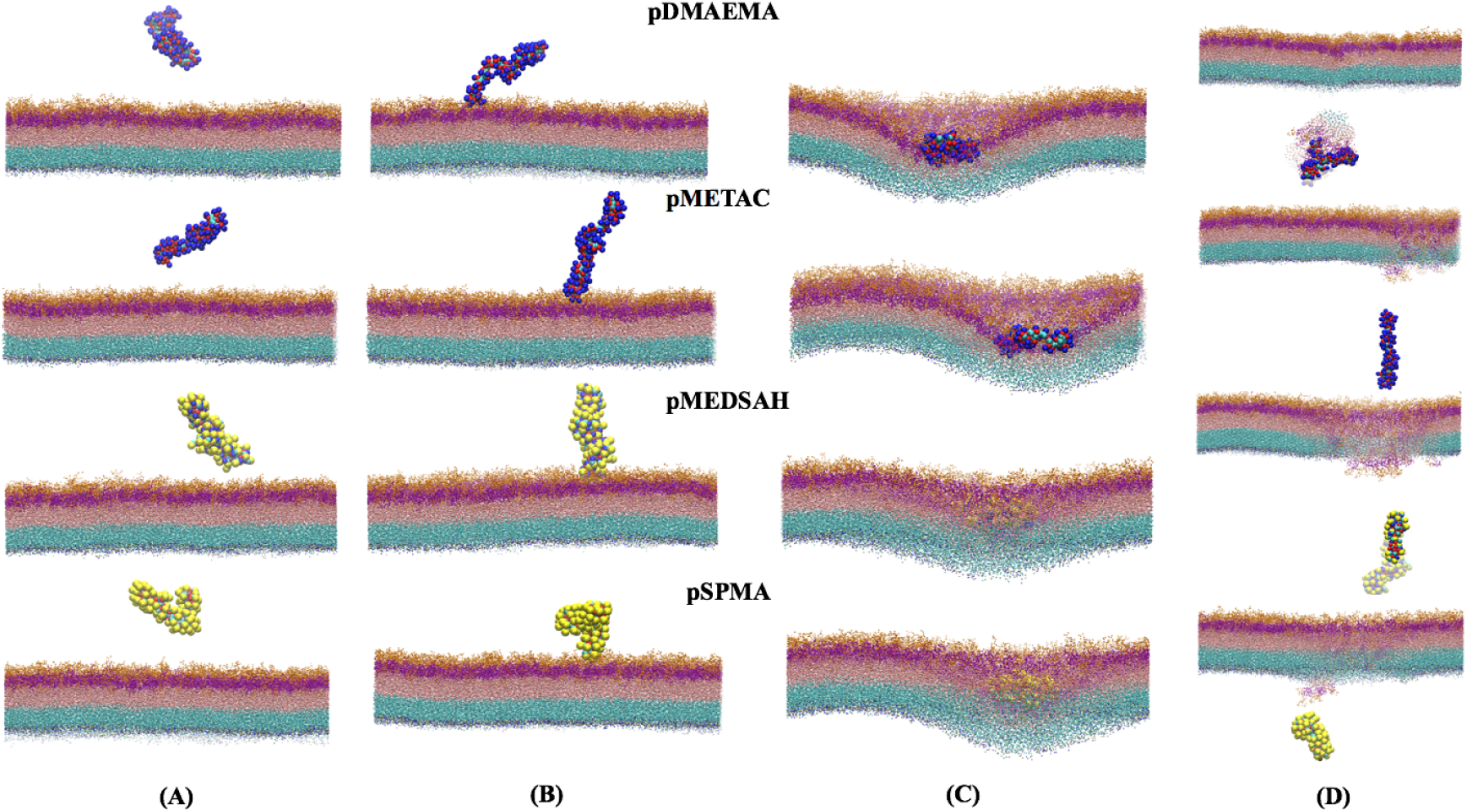
Steps of the polymer translocation through the bacterial outer membrane (OM) from steered molecular dynamics (SMD) simulations: approach (A), adhesion (B), permeation (C), and internalization (D).

The adhesion step was the most relevant for understanding the behavior of the different polymers at the interface with the bacterial OM and for the validation of the simulations against the experimental data (Figure 9B). In single-cell force spectroscopy experiments based on AFM measurements, this adhesion step approximates the attachment between the probe functionalized with a single immobilized bacterium and polymer brushes grown from glass surfaces.^31,61^ Force–distance curves that quantify the bacterial adhesion for a set of polymer brushes (pMETAC, pSPMA, and poly­(2-hydroxyethyl methacrylate) (PHEMA)) have been presented using this technique.^61^ The distinct magnitude of Δ*G*_adh_ for the cationic polymers (pDMAEMA and pMETAC) indicates a strong adhesion of these systems to the surface of the bacterial OM, which is in line with experimental data (Table 4).^12,31,61^ Furthermore, the highest *F*_adh_ values for the positively charged polymers also reflected the promoted bacterial adhesion in these systems compared to the remaining polymers (Table 3). The lower *F*_adh_ values (Table 3) and the less favorable Δ*G*_adh_ values of pMEDSAH and pSPMA are also in agreement with the low adhesion of proteins and bacteria to zwitterionic and anionic polymers, as reported in experimental studies (Table 4).^12,17,31,60^ For example, lysozyme has been shown to be repelled from zwitterionic brushes (poly­(carboxybetaine methacrylate), poly­(sulfobetaine methacrylate), and poly­((2-(methacryloyloxy)­ethyl)­phosphorylcholine)) as a result of the strong interactions of these polymers with water molecules.^17^ In addition to the presence of numerous pSPMA···Ca^2+^ interactions (Table 2), the anionic polymer (pSPMA) was the least desolvated during the adhesion step (Figure 6), which demonstrates its strong interaction with the solvent, thereby characterizing the bacterial-repelling activity of anionic polymers.^4^

The next step of the translocation mechanism of the polymers was the permeation step (Figure 9C), which exhibited substantial energetic barriers $\Delta G_{\mathrm{per}}^{‡}$ (Table 4), particularly for the cationic polymers (pDMAEMA and pMETAC), thereby illustrating the kinetically unfavorable character of this stage. This step was also characterized by pronounced, localized, and transient membrane defects (including local thinning and water penetration) induced by polymer penetration (Figure 9C). The transient, localized defect formation and the resistive behavior of this step were also reflected in the *F*_max_ values (Table 3) collected from the SMD simulations, which considered the full reaction coordinate for the translocation process (Figure 2E). The high number of interactions between the polymers and LPS molecules, and the absence of polymer-phospholipid interactions during the permeation step (Table 2), suggested that only the LPS molecules are dragged from the outer leaflet of the membrane to the inner leaflet. In particular, the smaller number of contacts for pSPMA···LPS (Table 2), the high number of pSPMA···Ca^2+^ contacts, and the higher intensity of the polymer–TW interactions (Figure 5), providing a bulky solvation shell around this anionic polymer, may also explain its lower binding affinity for the bacterial OM compared to the remaining systems.

The internalization step was the last stage of the translocation mechanism, in which the polymers leave the inner leaflet of the bacterial OM (Figure 9D). This step evidences the transient character of the permeation step, since the bacterial OM structure undergoes spontaneous reconstitution after polymer permeation. Both translocation trajectories and the absence of polymer···phospholipid interactions (Table 2) demonstrate that only LPS molecules are dragged by the polymers from the outer leaflet to the inner leaflet of the OM (Figure 9D). This behavior also resulted in the formation of nanopores on the membrane surface after the polymer translocation to the intracellular compartment of the membrane. For the cationic polymers, a high number of interactions with the LPS molecules was observed (Table 2), especially for the pDMAEMA, which reached the intracellular medium with an adsorbed LPS in its structure (Figure 9D). Hole formation in phospholipid membranes has also been reported in both *in silico* and *in vitro* studies involving the interaction of polycationic polymers with these membranes.^67,68^ The experimental studies point out that these membrane defects are able to trigger cell death.^67,68^ This effect may be enhanced in the presence of a polymer brush, where the array of chains could also promote the formation of pores in the structure of the bacterial OM. These membrane defects during the internalization step were also detected in the mass density profiles of the bacterial OM (Figure 3D) and Ca^2+^ (Figure 4D), which demonstrated the presence of water and Ca^2+^ ions at the center of the membrane after polymer translocation. Finally, both pMEDSAH and pSPMA were more hydrated than the cationic polymers in the internalization step (Figure 6), which was also in line with hydration data of zwitterionic and anionic polymers.^14,61–63,69^

At last, the simulations are in full agreement with the experimental observation that cationic polymers (pDMAEMA and pMETAC) strongly adhere to the bacterial OM. Moreover, the simulations indicate that the strong bacterial adhesion is governed by favorable electrostatic interactions, combined with the dragging of LPS molecules from the membrane during the translocation process. Indeed, one of the hypotheses regarding the bactericidal mechanism of these polymers involves bacterial OM disruption with the formation of holes that lead to the leakage of cell compounds and the consequent destabilization of this primary molecular barrier of the bacteria.^4,70^ However, a molecular-level understanding of such an effect is largely absent in the literature, though similar bacterial OM disruptive mechanisms have been reported for antimicrobial peptides.^71^ The simulations were able to access molecular details of the adhesion process of antibacterial and antifouling polymers on bacterial OM, highlighting the thermodynamics, kinetics, and dynamics of these interactions.

## 4 Conclusions

Polymer brushes are widely used as antibacterial and antifouling coatings to limit bacterial colonization, yet the molecular determinants that make some chemistries adhesive, while others remain repellent to Gram-negative bacteria, are still not fully resolved. Here, we used steered molecular dynamics and umbrella sampling to map the free-energy landscape for interactions between four methyl methacrylate-derived polymers (pDMAEMA, pMETAC, pMEDSAH, and pSPMA) and an *Escherichia coli* outer membrane model. The translocation pathway was resolved into four stages—approach, adhesion, permeation, and internalization—where approach and adhesion occurred spontaneously, whereas permeation and internalization were kinetically unfavorable due to large free-energy barriers. The thermodynamic driving force for adhesion follows pMETAC > pDMAEMA > pMEDSAH > pSPMA. Notably, the strong cationic pMETAC exhibits an Δ*G*_adh_ that is 175.7 kJ mol^–1^, 320.9 kJ mol^–1^, and 476.9 kJ mol^–1^ more favorable than pDMAEMA, pMEDSAH, and pSPMA, respectively, consistent with single-cell force spectroscopy measurements.^4^ In contrast, the unfavorable adhesion of the zwitterionic pMEDSAH and anionic pSPMA aligns with experimental evidence that zwitterionic and anionic brushes resist protein and bacterial attachment. Beyond ranking adhesion strength, the PMFs, together with ion/hydration signatures, provide a controlled thermodynamic basis to interpret why strongly cationic chemistries form deeper interfacial minima, whereas highly hydrated or charge-balanced chemistries remain weakly bound and more reversible without implying spontaneous full translocation under physiological conditions. In the context of grafted brushes, the relevant physics is governed by the distal, solvent-exposed portions of the tethered chains that first contact the OM; thus, the “wells” and “barriers” identified here should be interpreted as segment-accessible free-energy minima and insertion barriers that control the likelihood and persistence of membrane contact. Future work may build on this minimal framework by simulating multichain brush patches with controlled grafting density and crowding to assess how collective electrostatics and hydration modulate the polymer–OM free-energy landscape.

## Supplementary Material



## Data Availability

The structures, topologies, and input files are freely available for download at GitHub:https://github.com/BioMat-USP-RP/Input-files-for-CG-simulations-of-polymers-and-bacterial-outer-membrane.

## Notes

Temporal variation of the root-mean-square deviation (RMSD) and the radius of gyration (*R*_g_) of the free polymers pDMAEMA, pMETAC, pMEDSAH, and pSPMA; Average values of the RMSD and *R*_g_ of the free polymers; Time evolution of the area per LPS (*A*_LPS_), area per lipid of the phospholipids DPPE and DPPG (*A*_DPPE/DPPG_), and membrane thickness (*D*_HH_) of the bacterial OM; Average mass density profile of the OM; Average values of the *A*_LPS_, *A*_DPPE/DPPG_, and *D*_HH_; Average values of the *R*_g_ during the steps (approach, adhesion, permeation, and internalization) of the translocation mechanism of the polymers through the bacterial OM; Average values of the coordination number (CN) of water beads at a 4 nm radial distance from the center of mass of the polymers during the steps of the translocation mechanism of the polymers through the bacterial OM; Histograms of configurations corresponding to the windows sampled with the umbrella sampling (US) method along the reaction coordinate; Block analysis of the potentials of mean force (PMFs) (PDF)
