# Supplementary material for: Exploring the Interactions Between Methyl Methacrylate Polymers and the Bacterial Outer Membrane via Coarse-Grained Molecular Dynamics Simulations
Source: J Chem Inf Model. 2026 Apr 7;66(12):7138–53. doi: 10.1021/acs.jcim.6c00729 (PMC13292221; doi:10.1021/acs.jcim.6c00729)
Supplement: Supplementary file 1 [file ci6c00729_si_001.pdf]

Supporting Information

Exploring the Interactions between Methyl Methacrylate Polymers and the Bacterial Outer Membrane via Coarse-Grained Molecular Dynamics Simulations

Eduardo R. Almeida,[1,*] Vinicius Firmino dos Santos,[1] Madeleine Ramstedt,[2] Thereza A. Soares[1,3*]

[1]Departament of Chemistry, FFCLRP, University of São Paulo, Ribeirão Preto, SP 14040-901, Brazil.

[2]Department of Chemistry, Umeå University, Umeå 901 87, Sweden.

[3]Hylleraas Centre for Quantum Molecular Sciences, University of Oslo, Oslo 0315, Norway.

Table of Contents

Table S1. Chemical and physical properties of the studied methyl-methacrylate polymers. S3

Table S2. Composition of the coarse-grained bacterial outer membrane (OM) model of *Escherichia coli*. S3

Figure S1 Computational workflow conducted in this work using the following methods implemented in the GROMACS software version 2019.4:[8] steered molecular dynamics (SMD),[9] umbrella sampling (US) method,[10] and Weighted Histogram Analysis Method (WHAM).[11] S4

Figure S2 Time evolution of the root mean square deviation (RMSD) and the radius of gyration (R_g) of the free polymers pDMAEMA, pMETAC, pMEDSAH, and pSPMA in aqueous solution during the 1.0 μs simulations: RMSD (A) and R_g (B). S5

Table S3. Structural properties of the free polymers pDMAEMA, pMETAC, pMEDSAH, and pSPMA in aqueous solution calculated as average values from molecular dynamics simulations of 1.0 μs. S5

Figure S3 Temporal variation of the structural properties of the bacterial outer membrane (OM) model during the last 10 ns of the 6.0 μs molecular dynamics simulation (equilibration run): area per lipid of the LPS molecules (A), area per lipid of the phospholipids DPPE and DPPG (B), and membrane thickness (C). Average mass density profile (D). S6

Table S4. Average values of the structural properties of the bacterial outer membrane (OM) model calculated from the last 10 ns of the 6.0 μs molecular dynamics simulation (equilibration run) at 310 K and 1.0 bar. S7

Figure S4 Density profiles of the Cl^- and Na^+ ions during the translocation process of the polymers (pDMAEMA, pMETAC, pMEDSAH, and pSPMA) through the bacterial outer membrane (OM): approach step (A), adhesion step (B), permeation step (C), and internalization step (D). Each profile was calculated as an average from three independent runs. S7

Table S5. Coordination number (CN) of water beads (TW bead of the MARTINI 3 force field) at a 4 nm radial distance of the polymers (pDMAEMA, pMETAC, pMEDSAH, and pSPMA) during their translocation through the bacterial outer membrane (OM). S8

Figure S5 Configuration histograms corresponding to the simulation windows sampled with the umbrella sampling (US) method. These windows were collected from the reaction coordinate that describes the translocation processes of the polymers studied herein (pDMAEMA, pMETAC, pMEDSAH, and pSPMA) toward the center of the bacterial outer membrane (OM) model: pDMAEMA-OM (A), pMETAC-OM (B), pMEDSAH-OM (C), and pSPMA-OM (D). Count represents the number of configurations, whereas the reaction coordinate is the axis perpendicular to the plane of the OM. S8

Figure S6 Block analysis of the potentials of mean force (PMFs) that describe the translocation processes of the polymers (pDMAEMA, pMETAC, pMEDSAH, and pSPMA) toward the bacterial outer membrane (OM) center. S9

References S9

*Corresponding authors: almeida.eduardo@usp.br; thereza.soares@usp.br

Table S1. Chemical and physical properties of the studied methyl-methacrylate polymers.

Polymer	Properties				
	Ionic character[a]	Charge[b]	Ionizable group[a]	Hydrophilicity[c, d,e]	Volume/nm^3 [f]
pDMAEMA	cationic	+96	tertiary amine	hydrophilic	1356.6
pMETAC	cationic	+96	quaternary ammonium	highly hydrophilic	1487.6
pMEDSAH	zwitterionic	0	quaternary ammonium+ sulfonate	highly hydrophilic	1315.1
pSPMA	anionic	-96	sulfonate	hydrophilic	1218.8

[a]Ref.1. [b]The charge of these polyelectrolyte polymers corresponds to the sum of the individual charges of their constituent monomers. [c]Ref.2. [d]Ref.3. [e]Ref.4. [f]The volume of the polymers was estimated as the excluded volume of the solvent from the solvent assessment surface area (sasa) calculation implemented in GROMACS version 2019.4 (ref.5,6).

Table S2. Composition of the coarse-grained bacterial outer membrane (OM) model of *Escherichia coli*.

	Number of species[a]	
**Components**	**Outer leaflet**	**Inner leaflet**
rough LPS[b]	560	0
DPPE[c]	0	1260
DPPG[d]	0	420
**Ions**		
Ca^{2+} [e]	3012	
Cl^- [f]	4	
**Solvent**		
TW[g]	616312	

[a]All species were represented with the MARTINI 3 force field (FF) (ref.7). [b]Rough lipopolysaccharide parameterized as discussed in ref. 8. [c]1,2-dipalmitoyl-sn-glycero-3-phosphoethanolamine. [d]1,2-dipalmitoyl-sn-glycero-3-phospho-rac-glycerol. [e]The Ca^{2+} ion was modeled by the divalent bead CA (ref.

7). [f]The Cl^- ion was modeled by the monovalent bead (ref. 7). [g]The water molecules were modeled with the tiny bead water (ref. 7).

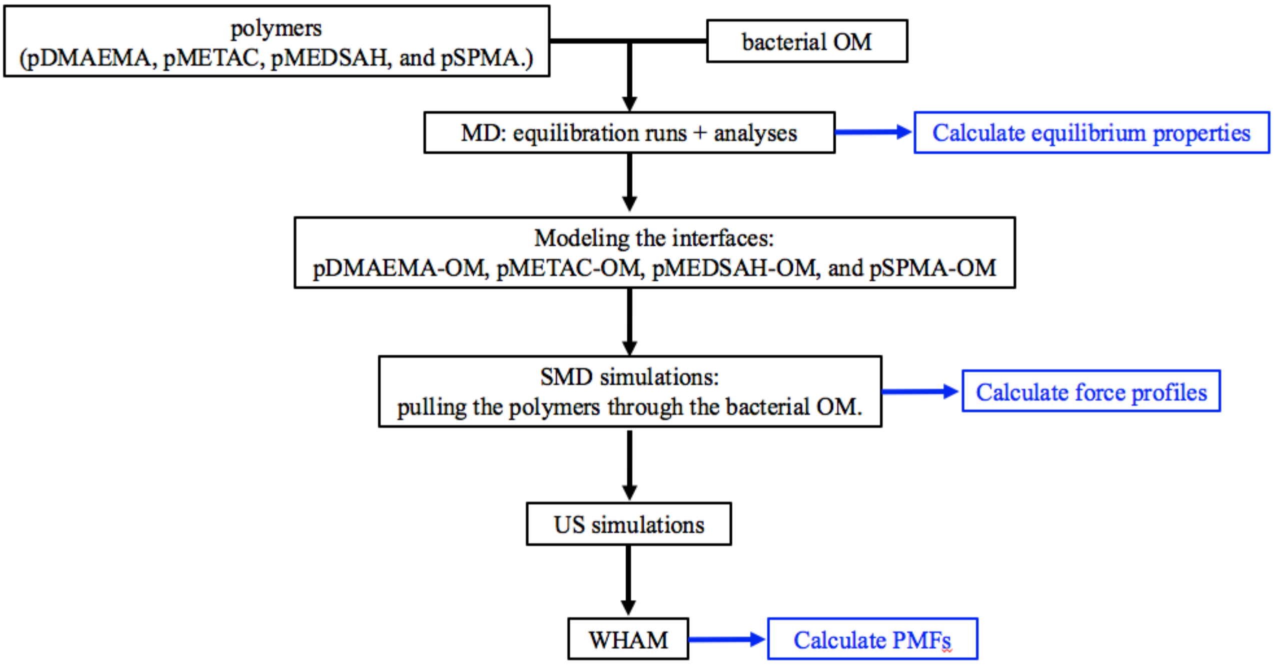

Figure S1 Computational workflow conducted in this work using the following methods implemented in the GROMACS software version 2019.4:[8] steered molecular dynamics (SMD),[9] umbrella sampling (US) method,[10] and Weighted Histogram Analysis Method (WHAM).[11]

Before analyzing the interaction between the free polymers and the bacterial OM, we firstly evaluated the equilibration of the isolated systems in aqueous solution at 310 K and 1 bar. Regarding the free polymers, the convergence in the time evolution of the root mean square deviation (RMSD) and the radius of gyration (R_g) in Figure S2 suggests structural stability of these systems during the 1.0 μs simulations.

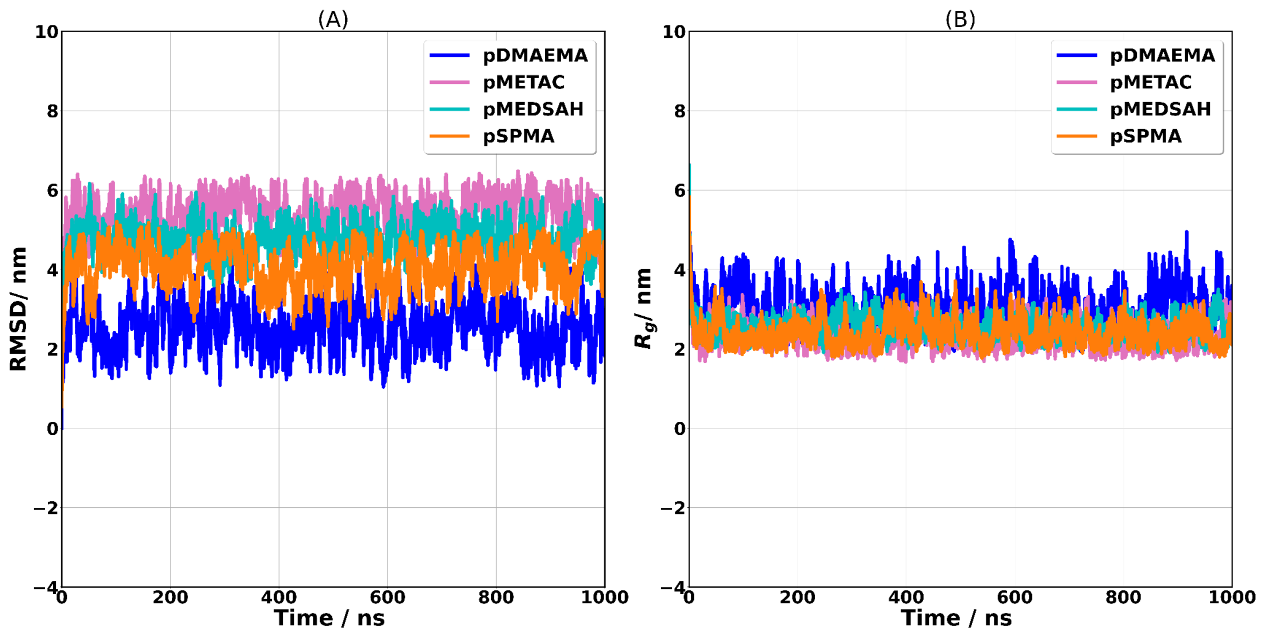

Figure S2 Time evolution of the root mean square deviation (RMSD) and the radius of gyration (R_g) of the free polymers pDMAEMA, pMETAC, pMEDSAH, and pSPMA in aqueous solution during the 1.0 μs simulations: RMSD (A) and R_g (B).

Data from Table S3 reinforce this equilibration since the standard deviation values of these measures represent about 13 % of the average RMSD and 13.3 % of the average R_g for all polymers. In particular, the R_g of the polymers pMETAC, pMEDSAH, and pSPMA indicates that the structures of these systems are more compact than the one of pDMAEMA with a difference of 0.63 nm in the R_g between these species. This behavior may be related to the higher molecular volume of pMETAC, pMEDSAH, and pSPMA and their charges that may have increased the number of solute-solute interactions favoring the level of compaction of these polymeric chains.

Table S3. Structural properties of the free polymers pDMAEMA, pMETAC, pMEDSAH, and pSPMA in aqueous solution calculated as average values from molecular dynamics simulations of 1.0 μs.

Polymer	RMSD / nm [a]	R_g / nm [b]
pDMAEMA	2.56±0.58	3.04±0.48
pMETAC	5.41±0.47	2.28±0.30
pMEDSAH	4.77±0.41	2.54±0.27
pSPMA	4.08±0.51	2.41±0.33

[a]Root mean square deviation. [b]Radius of gyration.

For the bacterial OM model, the stable behavior of the temporal variation of both area per lipopolysaccharide (A_{LPS}) and per DPPE and DPPG ($A_{DPPE/DPPG}$) in Figure S3A and Figure S3B suggests the convergence of these properties and, therefore, the structural stability of this asymmetric

bilayer after 1.0 μs. The average A_{LPS} per acyl chain (0.298 nm^2) is in agreement with the range of theoretical and experimental values reported per acyl tail (0.26-0.32 nm^2).[8,12-17] Finally, in addition to the stable time series of the membrane thickness in Figure S3C, the average mass density profile in Figure S3D also reflects the structural stabilization of this bacterial membrane prototype. In particular, although the solvent was also represented at the CG resolution, Figure S3D shows that the saccharides core of the LPSs and the polar heads of the phospholipids (DPPE and DPPG) are fully hydrated, which indicates that the tiny water beads (TW) of the MARTINI 3 force field[7] were able to properly represent the solvation of these molecules in agreement with the atomistic models.[14-16]

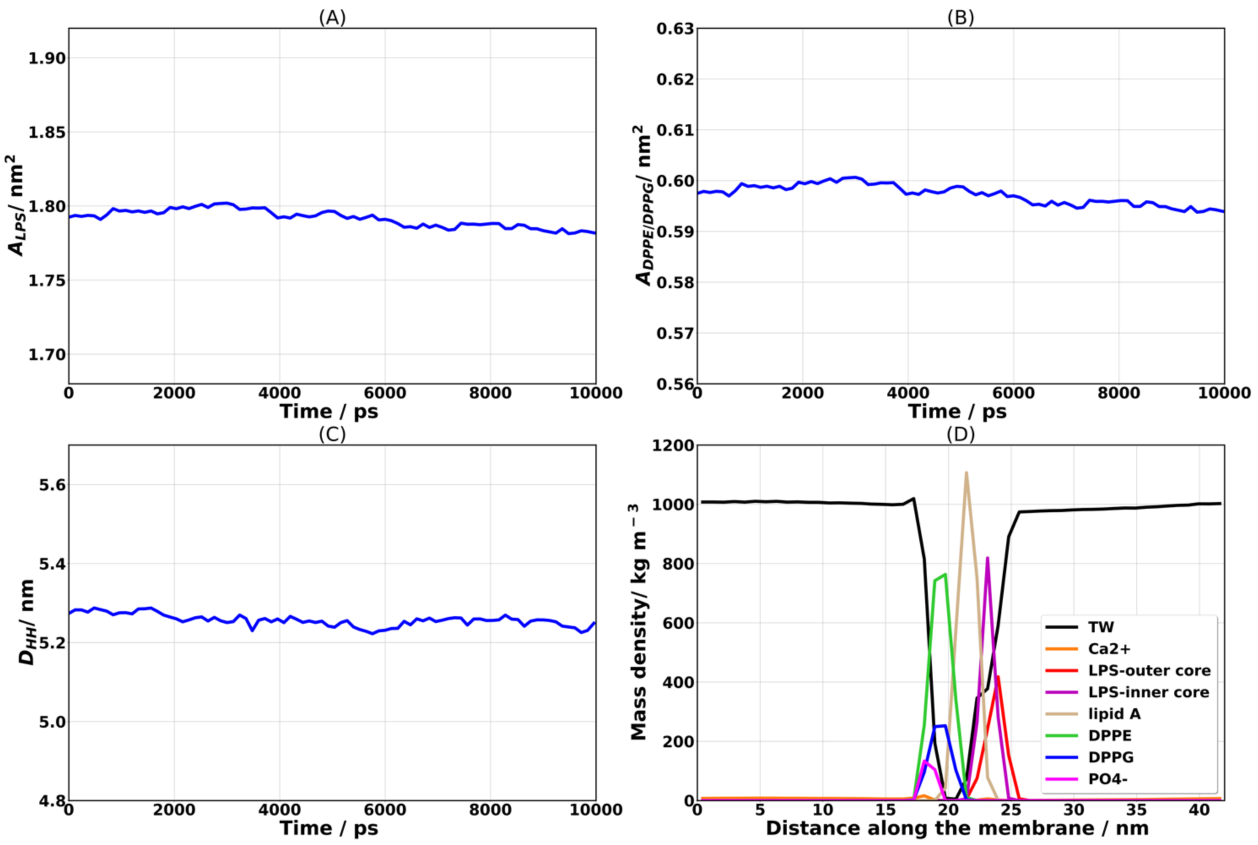

Figure S3 Temporal variation of the structural properties of the bacterial outer membrane (OM) model during the last 10 ns of the 6.0 μs molecular dynamics simulation (equilibration run): area per lipid of the LPS molecules (A), area per lipid of the phospholipids DPPE and DPPG (B), and membrane thickness (C). Average mass density profile (D).

Table S4. Average values of the structural properties of the bacterial outer membrane (OM) model calculated from the last 10 ns of the 6.0 μs molecular dynamics simulation (equilibration run) at 310 K and 1.0 bar.

**Property / unity**	**Average ± Std.**
A_{LPS} / nm^2 [a]	1.792 ± 0.006<br>(1.30-1.92)[d]
$A_{DPPE/DPPG} / nm^2$ [b]	0.597± 0.002<br>(0.605[e], 0.588[f], 0.55[g])
D_{HH} / nm [c]	5.257 ± 0.015

[a]Area per LPS molecule. [b]Area per lipid (DPPE and DPPG). [c]Membrane thickness is calculated as the average distance between the center of mass referring to the phosphates groups in the phospholipids and the center of mass referring to the outer core of the LPS molecules. [d]Ref. 7,12-17. [e]Ref. 18, value at 342 K for a homogeneous DPPE membrane.[f]Ref. 19, value at 333 K for a homogeneous DPPE membrane. [g]Ref. 20, value at 342 K.

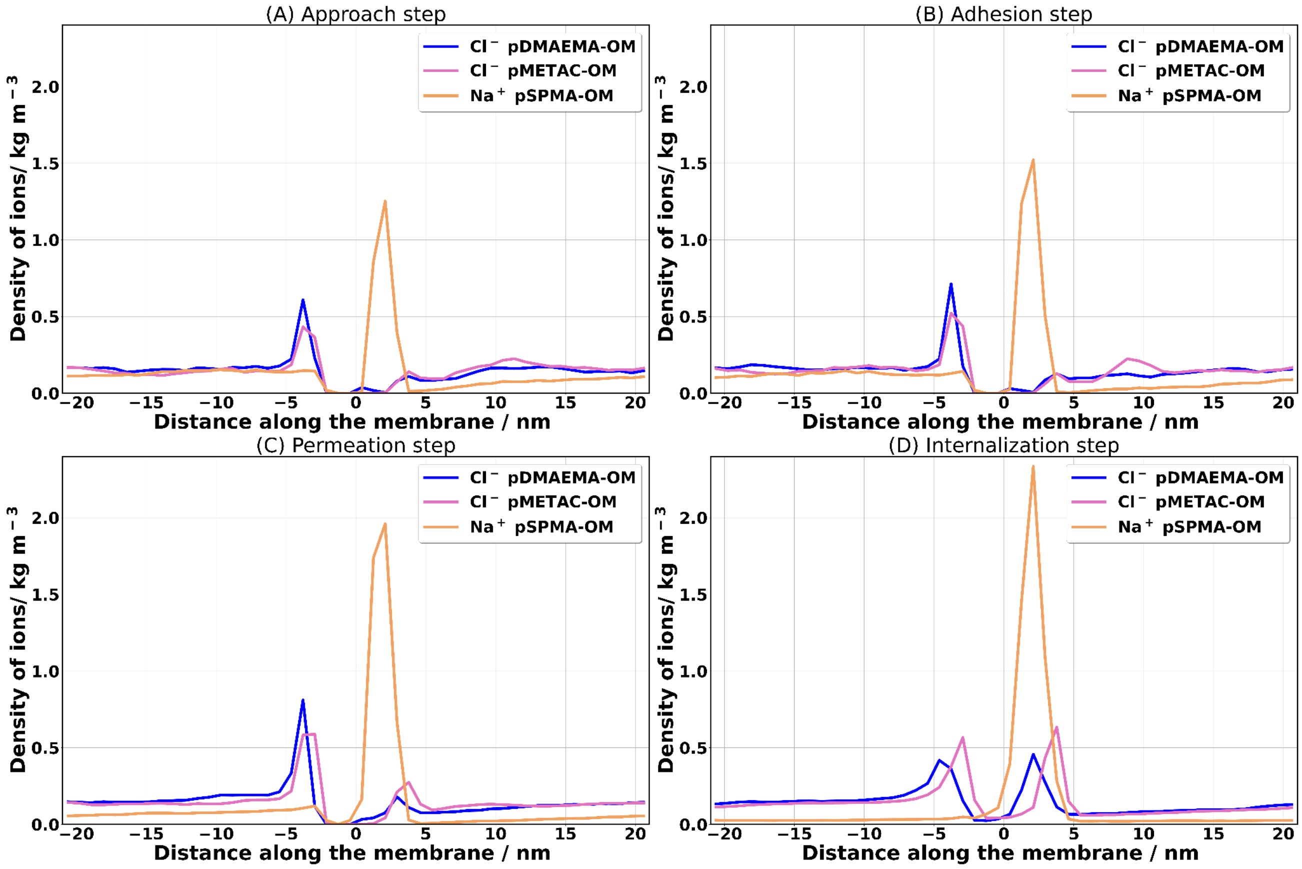

Figure S4 Density profiles of the Cl^- and Na^+ ions during the translocation process of the polymers (pDMAEMA, pMETAC, pMEDSAH, and pSPMA) through the bacterial outer membrane (OM): approach step (A), adhesion step (B), permeation step (C), and internalization step (D). Each profile was calculated as an average from three independent runs.

Table S5. Coordination number (CN) of water beads (TW bead of the MARTINI 3 force field) at a 4 nm radial distance of the polymers (pDMAEMA, pMETAC, pMEDSAH, and pSPMA) during their translocation through the bacterial outer membrane (OM).

System	CN(TW)*			
	**Approach**	**Adhesion**	**Permeation**	**Internalization**
pDMAEMA-OM	4295 ± 91	4016 ± 296	2993 ± 99	2987 ± 434
pMETAC-OM	4266 ± 15	4248 ± 8	3320 ± 89	3328 ± 52
pMEDSAH-OM	4147 ± 12	3852 ± 39	2978 ± 47	3747 ± 31
pSPMA-OM	4340 ± 19	4303 ± 7	3480 ± 24	3783 ± 15

*The CN was calculated from the radial distribution functions (g(r)) defined between the center of mass of the free polymers and the water beads (TW). Each value represents the average of three independent replicates.

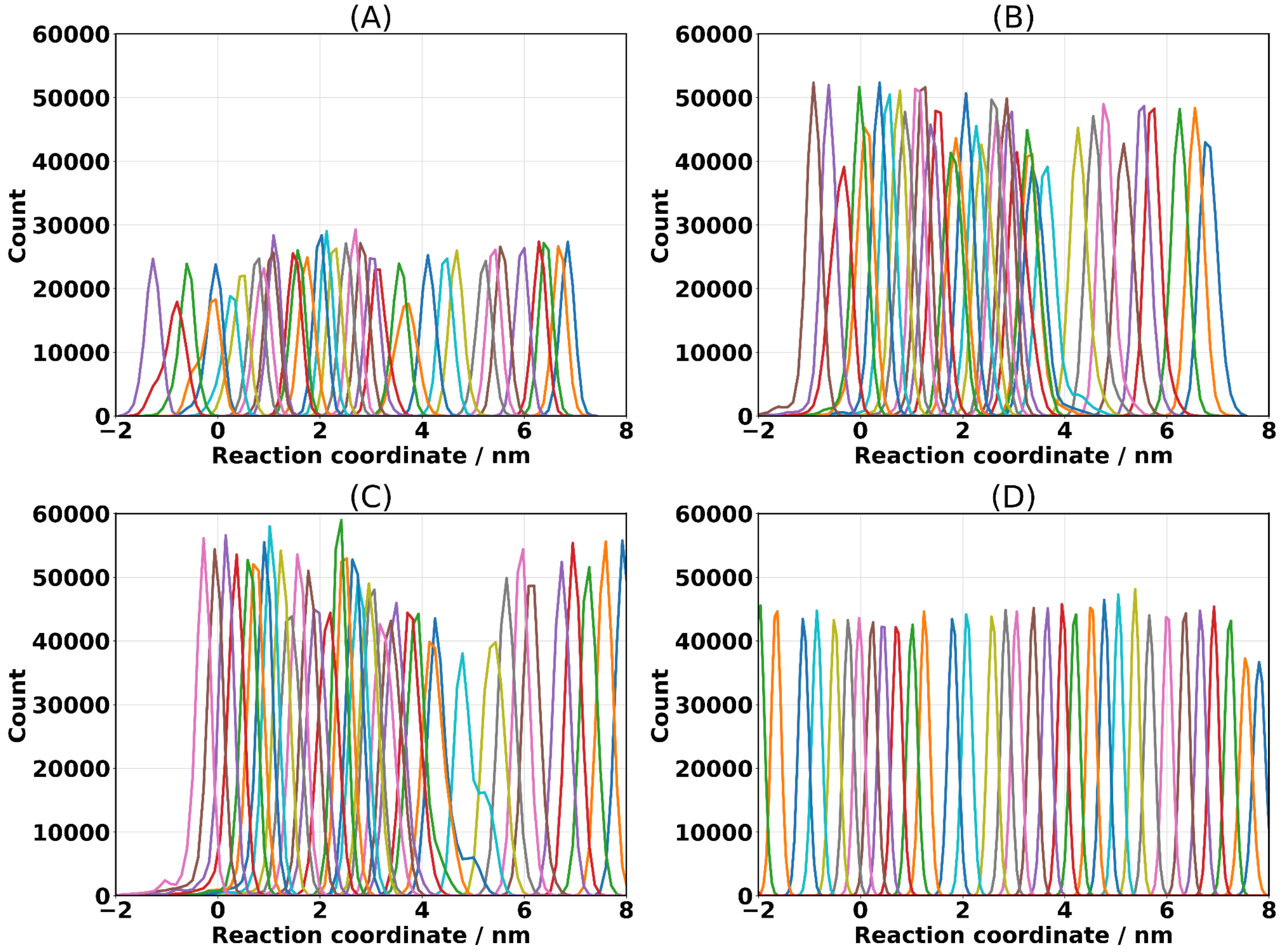

Figure S5 Configuration histograms corresponding to the simulation windows sampled with the umbrella sampling (US) method. These windows were collected from the reaction coordinate that describes the translocation processes of the polymers studied herein (pDMAEMA, pMETAC, pMEDSAH, and pSPMA) toward the center of the bacterial outer membrane (OM) model: pDMAEMA-OM (A), pMETAC-OM (B), pMEDSAH-OM (C), and pSPMA-OM (D). Count represents the number of configurations, whereas the reaction coordinate is the axis perpendicular to the plane of the OM.

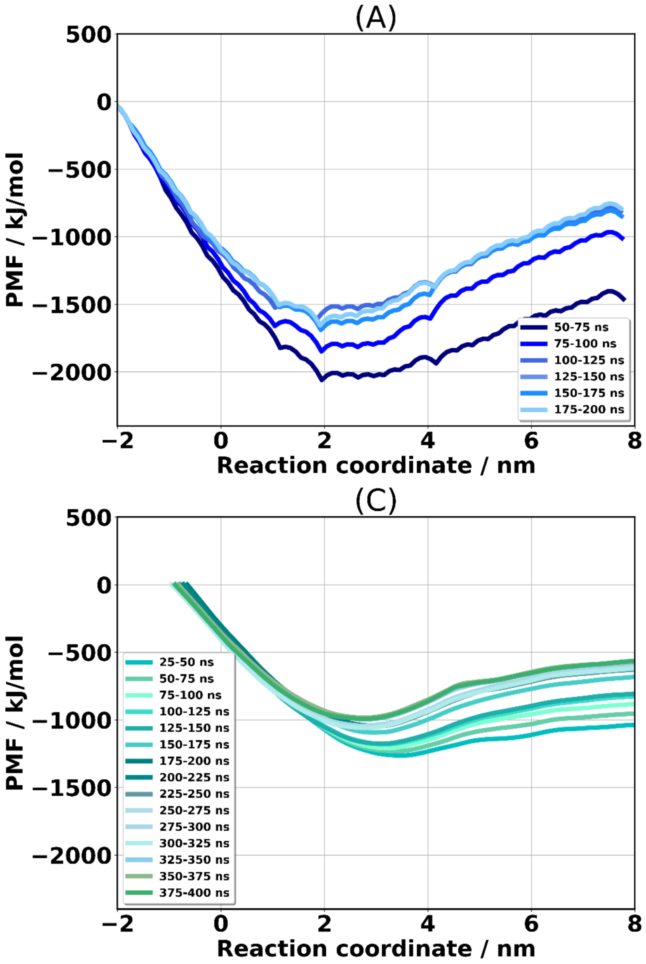

Figure S6 Block analysis of the potentials of mean force (PMFs) that describe the translocation processes of the polymers (pDMAEMA, pMETAC, pMEDSAH, and pSPMA) toward the bacterial outer membrane (OM) center.

References

1 Krishnamoorthy, M.; Hakobyan, S.; Ramstedt, M.; Gautrot, J. E. Surface-Initiated Polymer Brushes in the Biomedical Field: Applications in Membrane Science, Biosensing, Cell Culture, Regenerative Medicine and Antibacterial Coatings. Chem. Rev. **2014**, 114, 10976-11026, DOI: https://doi.org/10.1021/cr500252u

2 Bayliss, N.; Schmidt, B. V. K. J. Hydrophilic polymers: Current trends and visions for the future. Progress in Polymer Science **2023**, 147, 101753. DOI: https://doi.org/10.1016/j.progpolymsci.2023.101753

3 Jung, J. H.; Kang, Y. B.; Choi, C. Fabrication of Long-Lasting Superhydrophilic Anti-Fogging Film Via Rapid and Simple UV Process. Advanced Science **2025**, 12, 2409463. DOI: https://doi.org/10.1002/advs.202409463

4 Açari, I. K.; Sel, E.; Özcan, I.; Ates, B.; Köytepe, S.; Thakur, V. K. Chemistry and engineering of brush type polymers: Perspective towards tissue engineering. Adv. Colloid. Interface Sci. **2022**, 305, 102694. DOI: https://doi.org/10.1016/j.cis.2022.102694

5 Eisenhaber F.; Lijnzaad P.; Argos P.; Sander C.; Scharf, M. The double cubic lattice method: Efficient approaches to numerical integration of surface area and volume and to dot surface contouring of molecular assemblies. J. Comput. Chem. **1995,** 16, 273-284. DOI: https://doi.org/10.1002/jcc.540160303

6 Abraham, M. J.; Murtola, T.; Schulz, R.; Páll, S.; Smith, J. C.; Hess, B.; Lindahl, E. GROMACS: High performance molecular simulations through multi-level parallelism from laptops to supercomputers. SoftwareX. **2015**, 1-2, 19–25, DOI: https://doi.org/10.1016/j.softx.2015.06.001

7 Souza, P. C. T.; Alessandri, R.; Barnoud, J.; Thallmair, S.; Faustino, I.; Grünewald, F.; Patmanidis, I.; , Abdizadeh, H.; Bruininks, B. M. H.; Wassenaar, T. A.; Kroon, P. C.; Meler, J.; Nieto, V.; Corradi, V.; Khan, H. M.; Domański, J.; Javanainen, M.; Martinez-Seara, H.; Reuter, N.; Best, R. B.; Vattulainen, I.; Monticelli, L.; Periole, X.; Tieleman, D. P. T.; de Vries, A. H.; Marrink, S. J. Martini 3: A General Purpose Force Field for Coarse-Grained Molecular Dynamics. Nat. Methods **2021**, 18 (4), 382–388, DOI: 10.1038/s41592-021-01098-3

8 Vaiwala, R.; Ayappa, K. G. Martini‑3 Coarse-Grained Models for the Bacterial Lipopolysaccharide Outer Membrane of Escherichia coli. J. Chem. Theory Comput. **2024**, 20 (4), 1704-1716, DOI: 10.1021/acs.jctc.3c00471

9 Boubeta, F. M.; Garcia, R. M. C.; Lorenzo, E. M.; Boechi, L.; Estrin, D.; Sued, M.; Arrar, M. Lessons learned about steered molecular dynamics simulations and free energy calculations. Chem. Biol. Drug Des. **2019**, 93, 1129−1138. DOI: https://doi.org/10.1111/cbdd.13485

10 Kästner, J. Umbrella sampling. Wiley Interdiscip. Rev.: Comput. Mol. Sci. **2011**, 1, 932−942, DOI: 10.1002/wcms.66.

11 Kumar, S.; Rosenberg, J. M.; Bouzida, D.; Swendsen, R. H.; Kollman, P. A. The Weighted Histogram Analysis Method for Free-Energy Calculations on Biomolecules. I. The Method J. Comput. Chem. **1992**, 13, 1011, DOI: https://doi.org/10.1002/jcc.540130812

12 Snyder, S.; Kim, D.; McIntosh, T. J. Lipopolysaccharide bilayer structure: effect of chemotype, core mutations, divalent cations, and temperature. Biochemistry **1999**, 38, 10758−10767, DOI: https://doi.org/10.1021/bi990867d.

13 Brandenburg, K.; Funari, S. S.; Koch, M. H.; Seydel, U. Investigation into the Acyl Chain Packing of Endotoxins and Phospholipids under Near Physiological Conditions by WAXS and FTIR Spectroscopy. J. Struct. Biol. **1999**, 128, 175−186, DOI: https://doi.org/10.1006/jsbi.1999.4186.

14 Kirschner, K. N.; Lins, R. D.; Maass, A.; Soares, T. A. A Glycam-Based Force Field for Simulations of Lipopolysaccharide Membranes: Parametrization and Validation. J. Chem. Theory Comput. **2012**, 8, 4719-4731, DOI: https://doi.org/10.1021/ct300534j

15 Dias, R. P.; da Hora, G. C. A.; Ramstedt, M.; Soares, T. A. Outer Membrane Remodeling: The Structural Dynamics and Electrostatics of Rough Lipopolysaccharide Chemotypes. J. Chem. Theory Comput. **2014**, 10, 2488−2497, DOI: https://doi.org/10.1021/ct500075h

16 Nascimento Jr, A.; Pontes, F. J. S.; Lins, R. D.; Soares, T. A. Hydration, ionic valence and cross-linking propensities of cations determine the stability of lipopolysaccharide (LPS) membranes. Chem. Commun., **2014**, 50, 231-233, DOI: https://doi.org/10.1039/C3CC46918B

17 Sharma, P.; Parthasarathi, S.; Patil, N.; Waskar, M.; Raut, R J. S.; Puranik, M.; Ayappa, K. G.; Basu, J. K. Assessing Barriers for Antimicrobial Penetration in Complex Asymmetric Bacterial Membranes: A Case Study with Thymol. Langmuir **2020**, 36, 8800-8814, DOI: https://doi.org/10.1021/acs.langmuir.0c01124.
18 Petrache, H. I.; Dodd, S. W.; Brown, M. F.. Area per Lipid and Acyl Length Distributions in Fluid Phosphatidylcholines Determined by 2H NMR Spectroscopy. Biophys. J. **2000**, 79, 3172–3192, DOI: https://doi.org/10.1016/S0006-3495(00)76551-9.
19 Mulugu, T. R.; Thur,ond, R. L.; Alam, T. M.; Trouard, T. P.; Brown, M. Phospholipid headgroups govern area per lipid and emergent elastic properties of bilayers. Biophys. J. **2022**, 121(21), 4205-4220, DOI: 10.1016/j.bpj.2022.09.005.
20 Thurmond, R. L.; Dodd, S. W.; Brown, M. F. Molecular areas of phospholipids as determined by ^{2}H NMR spectroscopy. Comparison of phosphatidylethanolamines and phosphatidylcholines. Biophys. J. **1991**, 59, 108−113, DOI: 0006-3495/91/01/108/06.
